# Functionalized Docetaxel Probes for Refined Visualization of Mitotic Spindles by Expansion Microscopy

**DOI:** 10.1021/jacs.4c15608

**Published:** 2025-02-11

**Authors:** Gang Wen, Xiong Chen, Patrick Eiring, Volker Leen, Johan Hofkens, Markus Sauer

**Affiliations:** † Department of Biotechnology and Biophysics, Biocenter, University of Würzburg, Am Hubland, 97074 Würzburg, Germany; ‡ Department of Chemistry, KU Leuven, Leuven 3001, Belgium; § Chrometra Scientific, Kortenaken 3470, Belgium; ∥ Max Planck Institute for Polymer Research, 55128 Mainz, Germany; ⊥ Rudolf Virchow Center, Research Center for Integrative and Translational Bioimaging, University of Würzburg, Josef-Schneider-Str. 2, 97080 Würzburg, Germany

## Abstract

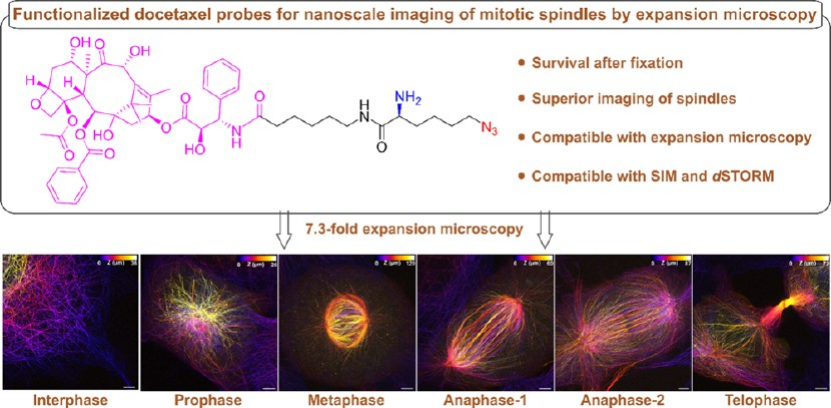

Visualizing the ultrastructure of mitotic spindles, the macromolecular machines that segregate chromosomes during mitosis, by fluorescence imaging remains challenging. Here we introduce an azido- and amino-functionalized docetaxel probe, which upon labeling of microtubules can be fixed, click-labeled and linked into hydrogels. The new probe is particularly useful for super-resolution imaging of dense microtubule structures in mitotic spindles. Multicolor expansion microscopy of mitotic cells allowed us to visualize the different phases of mitosis with unprecedented spatial resolution.

## Introduction

Mitosis is a fundamental process of cell division that is responsible for the accurate segregation of the duplicated genome into two daughter cells. In this process, the mitotic spindle, a highly intricate structure composed of microtubules, motor proteins and numerous other associated proteins, generates the forces required for the separation of sister chromatids between the two daughter cells.^1−3^ Mitosis is a precisely orchestrated process that relies on hundreds of diverse cellular proteins that ensure proper alignment, segregation, and movement of chromosomes. The precision with which chromosomes are aligned, segregated, and moved has intrigued scientists since the first description of chromosomes in the late 1800s.^4^ Mitosis involves the five phases prophase, prometaphase, metaphase, anaphase, and telophase that are followed by cytokinesis, the final stage of cell division, which is occasionally referred to as the sixth phase of mitosis.^5^ To understand the mechanisms underlying its functions in more detail improved visualization of the ultrastructure of mitotic spindles is necessary.

In recent years, various super-resolution microscopy methods allowed researchers to visualize the organization of cells and tissues at the nanoscale.^6^ However, visualizing the fine structure of mitotic spindles remained challenging due to the nonavailability of probes that enable dense labeling of microtubules in spindles required for super-resolution microscopy. Expansion microscopy (ExM) is a straightforward and cost-effective approach to achieve nanoscale imaging via physical expansion of biological samples, providing an ideal approach to resolve these dense and complex structures.^7,8^ Mitotic spindles have been successfully expanded and visualized using immunolabeling but the knowledge gain remained limited due to the low labeling density achieved that limited useful expansion factors.^9−11^ Particularly, immunolabeling with anti-tubulin antibodies shows poor and inhomogeneous labeling at the equator of anaphase and telophase spindles, where a dark zone is frequently observed.^12,13^ This is exacerbated when applying ExM, resulting in a discontinuous signal.

By binding to β-tubulin subunits of microtubules, fluorescent taxanes such as docetaxel stabilize microtubule structures in their polymerized form, providing another efficient strategy to image microtubules in living cells.^12,14,15^ Compared to immunolabeling, fluorescent docetaxel probes show effective labeling of dark zones in mitotic spindles.^12^ However, these probes are incompatible with ExM because docetaxel-microtubule complexes do not survive fixation. In addition, the common fixation conditions can also alter the structure of docetaxel-bound sites of microtubules resulting in the loss of binding ability although it was demonstrated that some taxanes, e.g., FLUTAX, show binding to very mildly fixed microtubules.^12^ Another yet not solved problem of taxane probes is their lack of anchorable groups for covalent cross-linking into polyacrylamide hydrogels during expansion resulting in efficient washing out of the fluorescence signal.

## Results and Discussion

**Figure 1:**
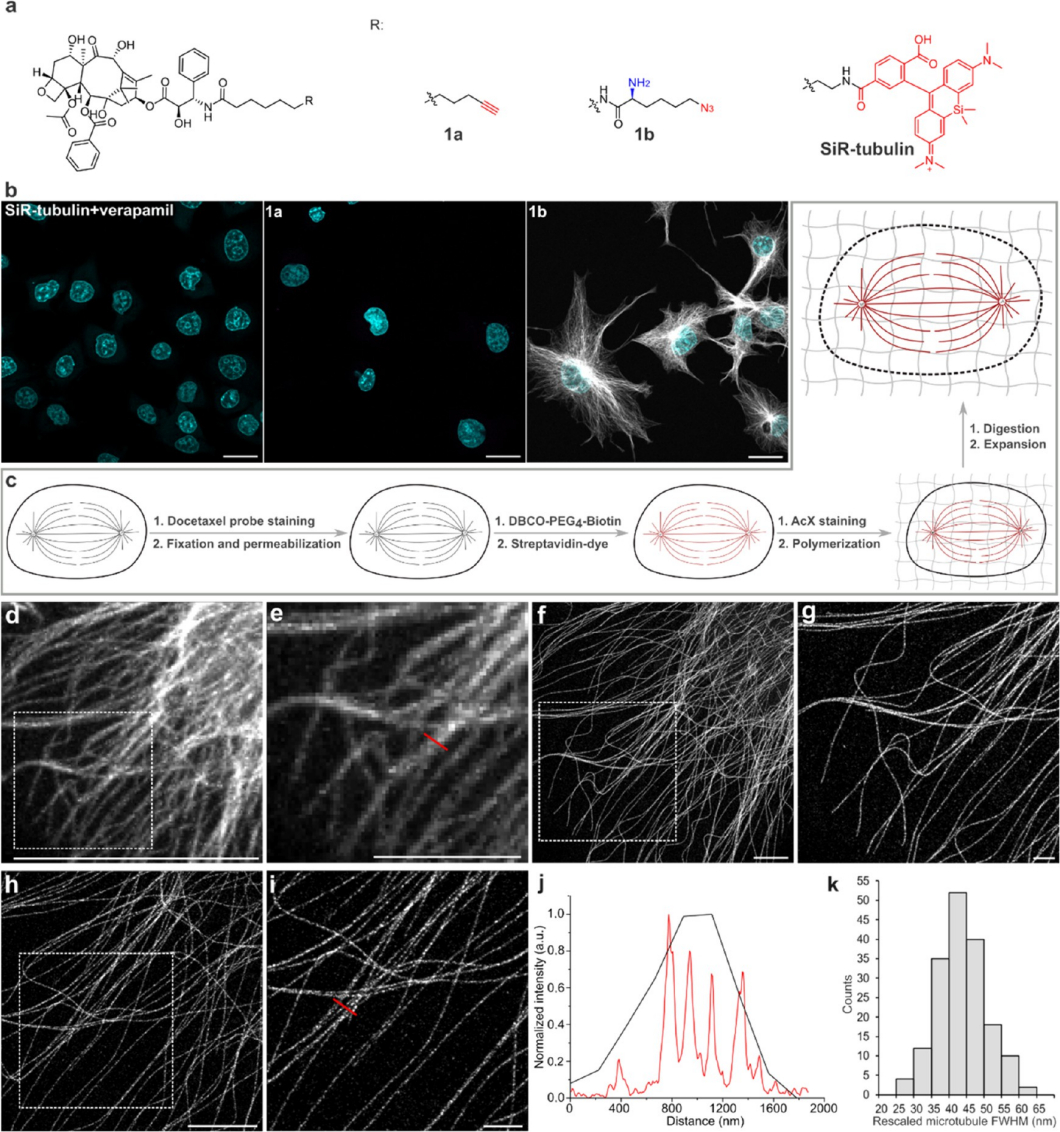
Concept and characterization of fixable docetaxel linkers. (a) Molecular structures of new docetaxel compounds **1a**, **1b** and SiR-tubulin. (b) Comparison of signal of microtubules stained with SiR-tubulin (left) or compound **1a**-**1b** (middle and right) in fixed cells. Cells labeled with the indicated probes were fixed with 0.5% GA and permeabilized with Triton X-100. ATTO 643-Azide and ATTO 643-DBCO were used to introduce fluorescence in samples labeled with compound **1a** or compound **1b**, respectively. DNA was stained with DAPI. All images were acquired with the same parameters. (c) Schematic of visualization of expanded mitotic spindles. After labeling microtubules with compound **1b**, cells are fixed, permeabilized, reacted with DBCO-PEG_4_-biotin and stained with streptavidin-dyes, followed by the procedure of ExM. (d–g) Comparison of confocal images of the same microtubules before (d, e) and after TREx (f, g). (h, i) Airyscan SR image of the same microtubules after TREx (h) and magnified views of the boxed region (i). (j) Line profiles along the red lines in (e, i). (k) Distribution of the full width at half-maximum (fwhm) values based on Gaussian fitting of intensity profiles of microtubules, yielding an average fwhm of 43.64 ± 7.14 nm (mean ± s.d., *n* = 173). Representative images were recorded from *n* > 4 (b) or 4 (d–i) independent samples. Scale bars, 30 μm (b, d, f, h), 10 μm (e, g, i).

To visualize details of mitotic spindles during different phases at higher spatial resolution, we developed a docetaxel analogue that can be cross-linked during fixation and is also compatible with ExM (Figure 1a). More specifically, we synthesized a docetaxel probe **1b** equipped with a primary amine moiety for fixation and an azido unit that enables the introduction of various fluorophores and functional groups via click chemistry after fixation. Due to the small size of the two functional groups introduced, alterations of the binding efficiency of the docetaxel derivative are unlikely. We also synthesized a docetaxel analogue **1a** carrying an alkyne group for comparison. First, we investigated the fixability of compound **1b** by comparing the fluorescence intensity of microtubules after fixation with the control experiment where SiR-tubulin,^14^ a commercially available live-cell microtubule marker was applied.

Here specimens stained with compound **1b** showed specific staining of microtubules after fixation with 0.5% glutaraldehyde (GA), permeabilization and click-labeling with fluorescent dyes while no signal of microtubules was detected for compound **1a**-stained samples or the control experiment with SiR-tubulin, indicating that the free amine supports fixation of the docetaxel probe (Figures 1b and S1–S2). This finding is consistent with a previously reported amino-containing ligand for membrane protein detection in fixed tissues.^16^ Next, we investigated the universal applicability of our new docetaxel probe for super-resolution imaging of microtubules in cells. Structured illumination microscopy (SIM) and airyscan microscopy images clearly show that various DBCO-functionalized organic dyes spanning the entire visible spectrum can be used for super-resolved microtubule imaging in cells (Figures S3–S4). *d*STORM images of microtubules demonstrate the superior performance of our new docetaxel probe. Microtubules are visualized as continuously labeled filaments with a diameter (fwhm) of 37.46 ± 3.62 nm (mean ± s.d., *n* = 10), highlighting the small linkage error of the probe (Figure S5). In addition, we also validated that our docetaxel probe allows specific labeling of microtubules in different cell lines and neurons (Table S1).

We then evaluated the performance of the functionalized docetaxel probe for imaging of microtubules by ExM. Therefore, COS-7 cells were labeled with probe **1b**, fixed and permeabilized, followed by click-reaction with DBCO-biotin and staining with CF568-modified streptavidin. Streptavidin enables efficient grafting of the probe into the polymer matrix, followed by digestion and expansion (Figure 1c). Using the 4× ExM protocol,^7^ we achieved an expansion factor of 4.3 by comparing the same microtubules pre- and postexpansion (Figure S6). Using airyscan microscopy, microtubules were imaged with an effective resolution of ∼66 nm (Figure S7).

To further improve the resolution, we tested the performance of probe **1b** in 10-fold robust expansion microscopy (TREx).^17^ Therefore, we optimized the anchoring efficiency by incubating the samples with Acryloyl-X SE (AcX) in sodium bicarbonate buffer. Using this protocol, we achieved an expansion factor of 7.3 for microtubules permitting fluorescence imaging with a resolution of 44 nm on an airyscan microscope (Figures 1d–k and S8). Furthermore, we exemplified the use of **1b** in multicolor ExM in combination with immunolabeling and small molecule-mediated labeling. Pre-expansion staining of the same cell first with compound **1b** followed by labeling with anti-α/β-tubulin antibodies demonstrates that the same cytoplasmic microtubules can be clearly visualized by TREx with high spatial resolution although a lower fluorescence intensity of astral microtubules was observed with the docetaxel probe (Figures S9–S11). Co-staining with antibodies directed against mitochondrial proteins and actin using trifunctional phalloidin linkers (the “Actin ExM” kit),^18,19^ enables the nanoscopic visualization of microtubules with mitochondria and actin, respectively, by TREx (Figures S12–S13).

Because of its performance, we hypothesized that our functionalized docetaxel probe might be ideally suited for microtubule imaging in mitotic spindles. And in fact, compound **1b** showed superior imaging performance when compared to immunolabeling. In particular, the docetaxel probe showed a bright microtubule signal at the spindle equator of anaphase cells, known as cleavage furrow and at the midbody part of telophase/cytokinesis cells, as well as near the spindle poles compared to dark or dim regions observed by immunolabeling, respectively (Figures S14–S15). We reasoned that efficient labeling of the midzone of mitotic cells is enabled by the small size of the functionalized docetaxel probe and fluorescently labeled streptavidin. The long distance and flexibility of the linker between biotin and docetaxel of more than 40 carbon/nitrogen/oxygen atoms ensures effective conjugation of biotin by streptavidin. Antibodies, in contrast, cannot access the binding epitopes in these dense regions possibly because of their larger size and associated steric hindrance.^12,13,20,21^

**Figure 2:**
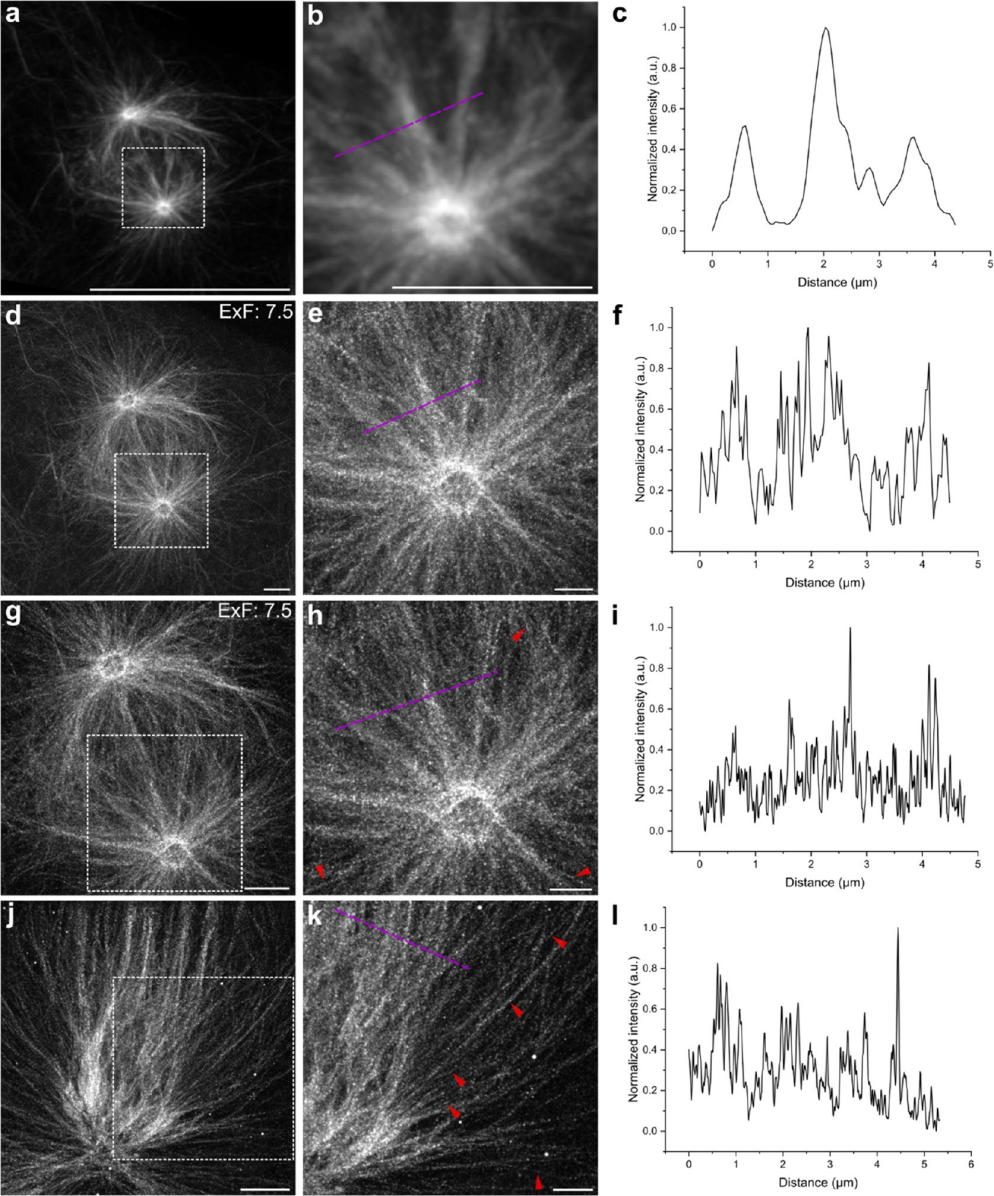
Comparison of super-resolution images of microtubules at mitotic states before and after TREx. (a) Pre-expansion airyscan image of microtubules. (b) Magnified view of the boxed region in (a). (c) Line profile along the purple dotted line in (b). (d) Post-expansion confocal image of microtubules in the same cell. (e) Magnified view of the highlighted region in (d). (f) Line profile along the purple dotted line in (e). The distance is corresponding to pre-expansion dimension based on expansion factor. (g) Airyscan microscopy image of the same microtubules after TREx. (h) Zoomed-in view of the boxed region in (g). The red arrowheads point at individual microtubules within microtubule bundles. (i) Line profile along the purple dotted line in (h). The distance is corresponding to pre-expansion dimension based on expansion factor. (j) Airyscan microscopy image of microtubules in the anaphase cell after TREx. (k) Zoom-in views of the highlighted region in (j). The red arrowheads point at individual microtubules within microtubule bundles. (l) Fluorescence intensity profiles along the purple dotted line in (k). In (f, i, l) the distance is corresponding to pre-expansion dimension based on expansion factor. Representative images were from three independent samples. Scale bars, 20 μm (a, d, g, j), 10 μm (e, h, k), 5 μm (b).

These results motivated us to apply the docetaxel probe for the visualization of mitotic spindles during different phases at higher spatial resolution using TREx. In these experiments, we first imaged the same spindle before and after expansion by confocal microscopy and determined an expansion factor of 7.5 (Figure 2a–f). Combining TREx with airyscan microscopy allowed us to image individual fibers in microtubule bundles at different mitotic stages (Figure 2g–l).

**Figure 3:**
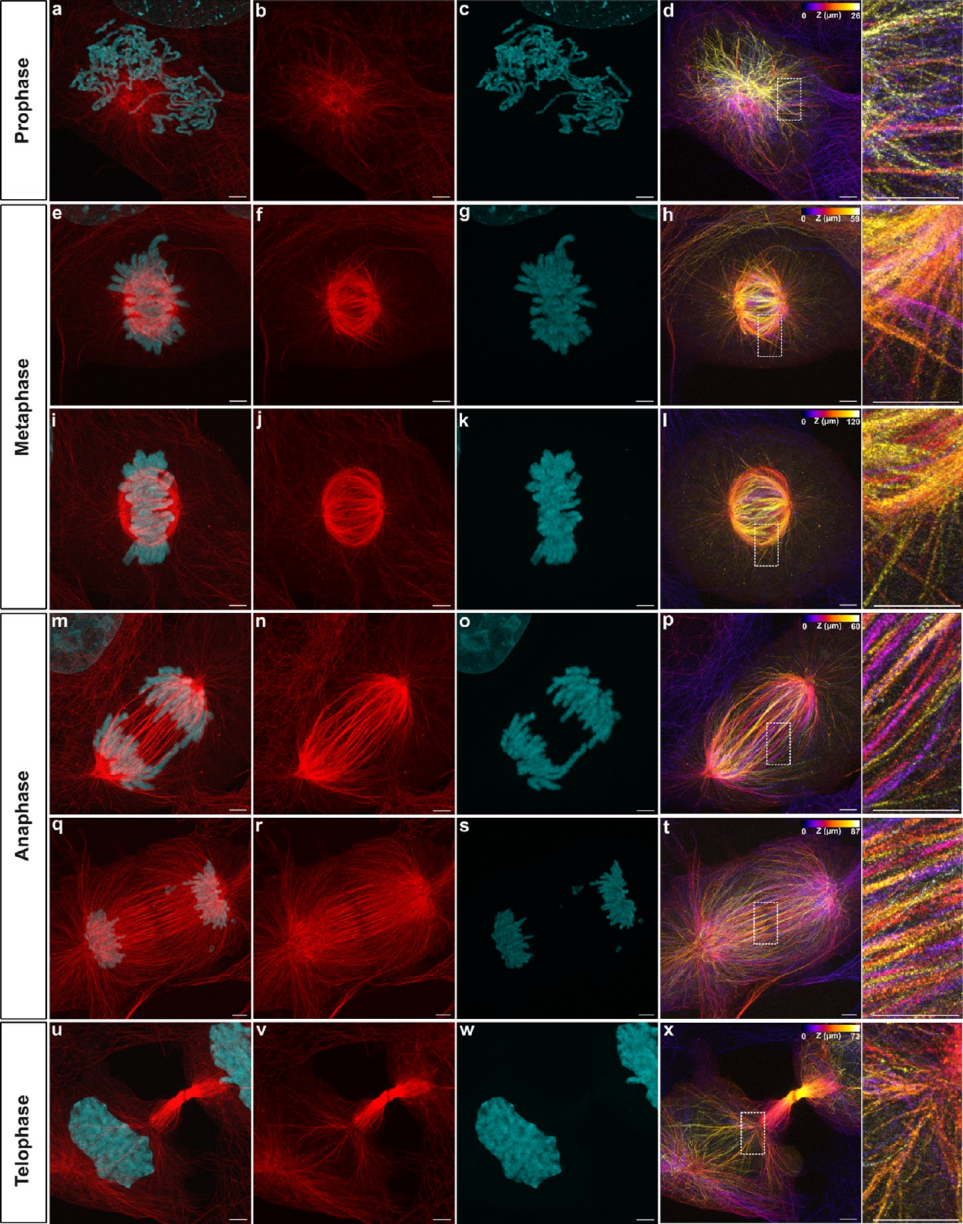
Super-resolution images of TREx-expanded microtubules at different mitotic stages. Microtubules in mitotic COS-7 cells were labeled with compound **1b**, fixed, permeabilized, click-labeled with DBCO-biotin, and stained with streptavidin-CF568, followed by expansion using the TREx protocol. DNA was stained with DAPI. (a–d) Two-color images of microtubules (red) and nuclei (cyan) at prophase (a–c) and corresponding 3D microtubule image (d). (e–l) Two-color images of microtubules (red) and nuclei (cyan) at metaphase (e–g, i–k) and corresponding 3D microtubule image (h, l). (m–t) Two-color images of microtubules (red) and nuclei (cyan) at anaphase (m–o, q–s) and corresponding 3D microtubule image (p, t). (u–x) Two-color images of microtubules (red) and nuclei (cyan) at telophase (u–w) and corresponding 3D microtubule image (x). The *z*-position is indicated by the color coding. Representative images were from three independent samples. Scale bars, 20 μm.

Co-staining experiments with anti-α/β-tubulin antibodies further demonstrated the superiority of the docetaxel labeling method. While immunolabeling showed a discontinuous signal of mitotic spindles, the docetaxel probe resolved fine details at the spindle equator, the midbody part and the spindle poles (Figures S16–S17). By staining DNA with DAPI, TREx ExM allowed us to resolve fine chromatin fibers of chromosomes in metaphase and anaphase cells (Figure 3). Using this imaging strategy, we were able to detect the interaction between microtubules and associated chromosomes at various mitotic stages with nanoscale resolution, including prophase (Figure 3a–d), metaphase (Figure 3e–l), anaphase (Figure 3m–t) and telophase (Figure 3u–x). Corresponding 3D images of mitotic spindles visualize details of the spatial arrangement of microtubules in these densely packed regions with superior spatial resolution (Figure 3d,h,l,p,t,x and Movies S1, S2, S3, S4, and S5).

## Conclusions

In conclusion, our small azido- and amino-functionalized docetaxel probe enables improved staining of dense microtubule structures in mitotic cells compared to standard immunolabeling. In combination with biotin click-labeling and fluorescently labeled streptavidin, it can be used advantageously in ExM to image mitotic spindles at different phases of mitosis with unprecedented spatial resolution. In addition, the docetaxel probe is compatible with other super-resolution microscopy methods such as SIM and *d*STORM thus representing a beneficial tool to unravel the precise mechanisms by which mitotic spindles regulate their architecture and morphometrics in different species.

## Experimental Section

### Cell Culture

COS-7 cells were cultured in DMEM/HAM’s F12 medium containing 10% fetal bovine serum (FBS), 100 U/mL penicillin and 0.1 mg/mL streptomycin in a humidified atmosphere containing 5% CO_2_ at 37 °C. Cells were seeded in 4-well Cellvis (Cellvis, # C 4–1.5H–N) at a concentration of 3 × 10^4^ cells/well or on a 12 mm coverslip (high precision, No. 1.5H) in 4-well plates at a concentration of 7 × 10^4^ cells/well for expansion experiments. The cells were then grown overnight.

### Microtubule Staining with SiR-Tubulin in Living Cells

The staining is performed based on instructions in the manufacturer’s manual. COS-7 cells were incubated with 1 μM SiR-tubulin in DMEM medium containing 10 μM verapamil and 1 μg/mL Hoechst 33342 for 60 min at 37 °C. Cells were then washed once with DMEM medium containing 10 μM verapamil prior to imaging.

### Microtubule Staining with Fixable Docetaxel Probes

COS-7 cells were fed with 3 μM of the docetaxel probe **1b** in DMEM medium for 1 h at 37 °C. Cells were then fixed with 0.5% GA in PEM solution (80 mM PIPES pH 6.8, 5 mM EGTA,1 mM MgCl_2_) containing 0.3% Triton X-100 for 10 min. After rinsing with phosphate-buffered saline (PBS), cells were quenched with freshly prepared 0.15% sodium borohydride in PBS at room temperature for 7 min. Cells were washed three times with PBS and treated with 20 mM *N*-ethylmaleimide for 30 min to block free thiols. Samples were further washed 3 times with PBS. Afterward, stained samples were incubated with 10 μM DBCO-modified dyes in PBS containing 1% BSA, followed by washing 3 times with PBS containing 0.1% Tween-20. For samples used in ExM, after blocking free thiols with *N*-ethylmaleimide, samples were incubated with 10 μM Biotin-PEG_4_-DBCO in PBS containing 1% BSA, followed by washing three times with PBS containing 0.1% Tween-20. Samples were then stained with 15 μg/mL CF568-modified streptavidin in PBS containing 1% BSA for 1 h, followed by washing three times with PBS. Finally, cells were incubated with 1 μg/mL DAPI for 1 min and washed three times again with PBS.

### Optimization of Microtubule Staining for *d*STORM

After incubating cells with 3 μM of the docetaxel probe in DMEM medium for 1 h at 37 °C, cells were fixed as mentioned above. Samples were treated with 20 mM *N*-ethylmaleimide and washed 3 times with PBS. The cells were then incubated with 2 μM DBCO-AF 647 in PBS at room temperature for 50 min, followed by washing 3 times with 0.1% Tween-20 in PBS. Afterward, samples were treated with 5% BSA for 20 min and immediately postfixed with 4% PFA for 10 min. Samples were quickly rinsed 3 times with PBS before image acquisition.

### Multicolor Staining

For costaining with antibodies to α/β-tubulin, after docetaxel-microtubule staining, cells were treated twice with blocking buffer (PBS, 1% BSA) for 5 min, followed by incubation with 5 μg/mL primary antibody (rabbit anti-α-tubulin, Abcam, ab18251 or mouse anti-β-tubulin, Merck, T8328) in staining buffer (PBS, 3% BSA) for 1 h at room temperature. The samples were washed 3 times with PBS and immunostained with AF 488-labeled secondary antibodies (goat anti-rabbit F(ab)_2_ IgG, ThermoFisher, A-11070 or goat anti-mouse IgG, Abcam, ab150113) in staining buffer for 1 h with a dilution of 100×. After rinsing twice with PBS, samples were washed twice with blocking buffer. Samples were then stained with 1 μg/mL DAPI for 1 min and washed three times again with PBS prior to imaging. For costaining with antibodies to mitochondria, after incubating cells with the docetaxel probe, cells were fixed with 0.2% GA in PEM solution containing 0.1% Triton X-100 for 10 min. Cells were then rinsed once with PBS and quenched with 0.15% sodium borohydride in PBS for 7 min. After washing 3 times with PBS, cells were further permeabilized using 0.2% Triton X-100 for 10 min and washed 3 times using PBS. Cells were stained with CF568-modified streptavidin as above-mentioned. Next, samples were then blocked with permeabilization/blocking buffer (PBS, 3% BSA and 0.25% Triton X-100) for 20 min, followed by staining with primary antibodies (rabbit anti-TOMM20, Abcam, ab186734) in staining buffer at a concentration of 4 μg/mL for 1 h at room temperature. After washing 3 times with PBS, samples were then incubated with AF 488-labeled secondary antibodies (goat anti-rabbit F(ab)_2_ IgG, ThermoFisher, A-11070) in staining buffer for 1 h with a dilution of 100×. Samples were then rinsed twice with PBS and washed twice with blocking buffer. Finally, samples were stained with DAPI as mentioned above. For costaining with actin filaments, the docetaxel-microtubule staining was performed as mentioned above, except that microtubules were labeled with 10 μg/mL ATTO 643-streptavidin. Next, actin filaments were stained with the trifunctional phalloidin linker (the “Actin ExM” kit, fluorophore 561) in PBS at a concentration of 0.5 μM at room temperature for 1 h, followed by washing three times with PBS. The immunostained samples were then treated with DAPI prior to imaging.

### Gelation, Digestion, and Expansion

The procedure of ExM was performed as previously described with some modifications.^7,17^ For 4× ExM, samples were first incubated with 0.1 mg/mL AcX in PBS for 90 min at room temperature and washed three times with PBS for 5 min. Afterward, samples were quickly rinsed with 50 μL monomer solution (2 M NaCl, 8.625% (w/w) sodium acrylate, 2.5% (w/w) acrylamide, 0.15% (w/w) *N*,*N*′-methylenebis(acrylamide) and 0.01% 4-hydroxy-TEMPO in PBS) containing 0.15% tetramethylenediamine (TEMED, w/w) and 0.15% ammonium persulfate (APS, w/w), followed by polymerization on the gelation chamber using another 50 μL gelation solution in a humidified incubator at 37 °C for 90 min. Samples were then digested with proteinase K (New England Biolabs) at a concentration of 8 units/mL in digestion buffer (50 mM Tris (pH 8.0), 1 mM EDTA, 0.5% Triton X-100, 0.8 M guanidine HCl) at 37 °C for 3 h. For TREx, samples were incubated with 0.1 mg/mL AcX in PBS containing 200 mM NaHCO_3_ for 90 min at room temperature and washed 3 times with PBS for 5 min prior to the gelation. The stained samples were quickly rinsed with 50 μL monomer solution (1.1 M sodium acrylate, 2.0 M acrylamide, 90 μg/mL *N*,*N*′-methylenebis(acrylamide) in PBS) supplemented with 1.5 mg/mL APS, 1.5 mg/mL TEMED, and 15 μg/mL 4-hydroxy TEMPO, followed by polymerization on the gelation chamber with another 50 μL gelation solution in a humidified incubator at 37 °C for 1 h. Samples were then digested with proteinase K (New England Biolabs) at a concentration of 8 units/mL in digestion buffer (50 mM Tris (pH 8.0), 1 mM EDTA, 0.5% Triton X-100, 0.8 M guanidine HCl) at room temperature for 10 h. Samples were digested with proteinase K (New England Biolabs) at a concentration of 8 units/mL in digestion buffer (50 mM Tris (pH 8.0), 2 mM CaCl_2_,0.5% Triton X-100, and 0.8 M guanidine HCl) at 37 °C for 10 h when the costaining with antibodies to anti-α/β tubulin was performed. The digested samples were restained with 1 μg/mL DAPI in PBS for 1 min and washed three times with 1× PBS. Finally, the digested gels were expanded with deionized H_2_O for 10 min. This expansion step was repeated four times with H_2_O until gels were fully expanded.

### Expanded Gel Stabilization

To minimize the gel drafting during image acquisition, the expanded gels were placed onto a one-well chambered plate (Cellvis, Product #: C1–1.5H–N) coated with poly-d-lysine. Briefly, the plate was incubated with 0.1% poly-d-lysine (w/v, 2 mL) at 37 °C for 30 min, followed by rinsing three times with 2 mL deionized H_2_O. The coated plate was then left to air-dry at room temperature. Afterward, the fully expanded samples were placed onto the plate for imaging.

### Microscopy and Image Analysis

Airyscan confocal or SR images were obtained on a LSM 900 with Airyscan 2 (Zeiss) system using a C-Apochromat 40×/1.2 numerical aperture (NA) water-immersion objective. For various organic dyes applied in the paper, excitation wavelengths and filter settings for various dyes were chose using the integrated dye presets in the ZEN 2 blue software (version 3.5) from Zeiss. The standard strength setting of 3D Airyscan processing was used to process the Airyscan SR data. SIM images were acquired on an Elyra 7 (Zeiss) equipped with four excitation lasers, a 405 nm diode (50 mW), a 488 nm OPSL (500 mW), a 561 nm OPSL (500 mW) and a 642 nm diode laser (500 mW). Imaging was performed using a Plan-Apochromat 63×/1.4 NA oil DIC M27 objective. Laser emissions were filtered by band-pass (BP) 570–620, long pass (LP) 655, BP 420–480, or BP 495–550. SIM processing was carried out using the Zeiss ZEN 3.0 SR FP2 (black) software. *d*STORM images were recorded using an oil immersion objective (Olympus APON 60xO TIRF, NA 1.49) on a home-built widefield setup equipped with an inverted microscope (Olympus IX-71). The system is equipped with excitation lasers of the wavelengths 639 nm (Genesis MX639–1000, Coherent), 558 (Genesis MX561–500, Coherent), 514 nm (Genesis MX514–500, Coherent) and 405 nm (iBeam-smart-S 405-S, TOPTICA). A dichroic beam splitter (ZT405/514/635rpc) was applied to separate the excitation beam from the fluorescence and the emission was further filtered through an emission filter Brightline HC 679/41 (Semrock) before being detected by the EMCCD-cameras (iXon Ultra 897, Andor). *d*STORM imaging was acquired in 100 mM MEA solution (pH 7.4) with an integration time of 20 ms for 30,000 frames, using laser intensities of ∼3 kW/cm^2^ and additional pulsing with UV light (405 nm). Acquired image stacks were reconstructed with rapidSTORM 3.3.^22^ After image acquisition, all Airyscan confocal and SR data were analyzed with ImageJ.^23^ Expansion factors were determined by comparing the same features of COS-7 cells before and after expansion. The image resolution was measured with ImageJ “FWHM_line” plugin with the Gaussian fitting and recalculated by the expansion factor.

## Supporting Information Available

Detailed experimental procedures, compound characterization data, including spectra of NMR, staining performance in different cell lines, and additional fluorescence microscopy methods, data, and controls (PDF)
3D animation of expanded microtubules at prophase shown in Figure 3a–d (Video S1) (MP4)
3D animation of expanded microtubules at metaphase shown in Figure 3i–l (Video S2) (MP4)
3D animation of expanded microtubules at anaphase shown in Figure 3m–p (Video S3) (MP4)
3D animation of expanded microtubules at anaphase shown in Figure 3q–t (Video S4) (MP4)
3D animation of expanded microtubules at telophase shown in Figure 3u–x (Video S5) (MP4)
