# Supplementary material for: Functionalized Docetaxel Probes for Refined Visualization of Mitotic Spindles by Expansion Microscopy
Source: J Am Chem Soc. 2025 Feb 11;147(8):6604–11. doi: 10.1021/jacs.4c15608 (PMC11869276; doi:10.1021/jacs.4c15608)
Supplement: Supplementary file 1 — ja4c15608_si_001.pdf [file ja4c15608_si_001.pdf]

Supporting Information

Functionalized docetaxel probes for refined visualization of mitotic spindles by expansion microscopy

Gang Wen*[1], Xiong Chen[2], Patrick Eiring[1], Volker Leen[3], Johan Hofkens[2,4] & Markus Sauer*[1,5]

[1]Department of Biotechnology and Biophysics, Biocenter, University of Würzburg, Am Hubland, 97074 Würzburg, Germany

[2]Department of Chemistry, KU Leuven, Leuven 3001, Belgium

[3]Chrometra Scientific, Kortenaken 3470, Belgium

[4]Max Planck Institute for Polymer Research, 55128 Mainz, Germany

[5]Rudolf Virchow Center, Research Center for Integrative and Translational Bioimaging, University of Würzburg, Josef-Schneider-Str. 2, 97080 Würzburg, Germany

Corresponding authors: G.W. (gang.wen@uni-wuerzburg.de) and M.S. (m.sauer@uni-wuerzburg.de)

Table of Contents:

1. Synthesis and Characterization 3

2. Figure S1. Comparison of average fluorescence intensity of microtubules obtained after fixation with different concentrations of GA 9

3. Figure S2. Evaluation of the fixability of SiR-tubulin 10

4. Figure S3. SIM image of microtubules in COS-7 cells stained with compound 1b and click-labeled with various organic dyes 11

5. Figure S4. Airyscan microscopy images of microtubules in COS-7 cells with probe **1b** and various organic dyes 12

6. Figure S5. *d*STORM image of microtubules in COS-7 cells using compound **1b** 13

7. Table S1. Microtubule staining in different cell lines with compound **1b** 14

8. Figure S6. Expansion factor determination using the original ExM protocol 15

9. Figure S7. Microtubules visualized by 4x-ExM-Airyscan SR 16

10. Figure S8. Expansion factor calculation for microtubules expanded using the TREx protocol 17

11. Figure S9. Comparison of fluorescence signal of astral microtubules stained with different strategies 18

11. Figure S10. Multi-color images of microtubules stained with compound **1b** and anti-β tubulin antibodies in TREx 19

12. Figure S11. Multi-color images of microtubules stained with compound **1b** and anti-α tubulin antibodies in TREx 20

13. Figure S12. Multi-color images of compound **1b**-labeled microtubules and immunostained mitochondria in TREx 21

14. Figure S13. Multi-color images of compound **1b**-labeled microtubules and actin filaments stained with phalloidin in COS-7 cells using TREx 22

15. Figure S14. Comparison of microtubules labeled in COS-7 cells with compound **1b** and anti-β tubulin antibodies at different cell cycle stages by airyscan microscopy 23

16. Figure S15. Comparison of microtubules labeled in COS-7 cells with compound **1b** and anti-α tubulin antibodies at different cell cycle stages by airyscan microscopy 24

17. Figure S16. Comparison of TREx-expanded microtubules in mitotic COS-7 cells using compound **1b** and antibodies to β tubulin 25

18. Figure S17. Comparison of TREx-expanded microtubules in mitotic COS-7 cells using compound **1b** and antibodies to α-tubulin 26

19. ^{1}H and ^{13}C NMR spectra 27

20. Reference 31

1. Synthesis and Characterization

1.1 General

Chemicals were purchased from Sigma-Aldrich, TCI, ACROS or Jena Bioscience. Dry solvents were used without further purification. Reactions were monitored with thin layer chromatography (TLC) with silica gel plates (Kieselgel 60 F254 plates, Merck) under UV light. 70-230 mesh silica 60 (E. M. Merck) was used to purify compounds on column chromatography. Mass spectra was obtained on a Shimadzu LC-MS 2020 Liquid Chromatograph Mass Spectrometer (ShimPack Gist C18 2 μm, 2.1x100 mm). ^{1}H NMR and ^{13}C NMR spectra were acquired on a Bruker Avance 400 MHz or a Bruker Avance II+ 600 MHz spectrometer using MeOD-d_4 or $CDCl_3$ as a solvent.

1.2 Synthetic procedures

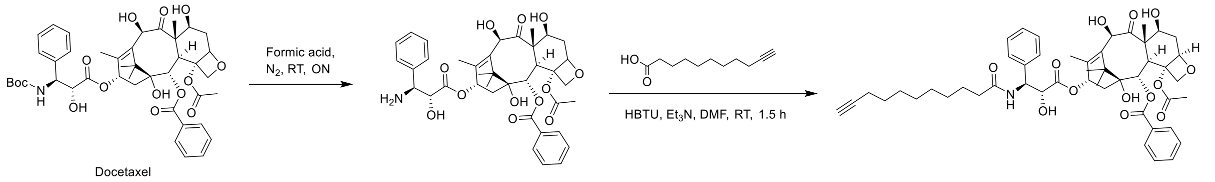

Synthesis of compound S1

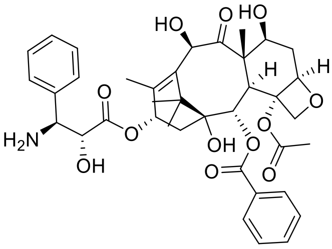

Docetaxel (100 mg) was dissolved in 0.5 mL formic acid and the reaction mixture was stirred for 3 h at room temperature. After complete reaction, all solvents were evaporated to yield the intermediate as a white solid, which was used without further purification. LC-MS (ESI$^+$): calculated for $C_{38}H_{46}NO_{12}$ $[M+H]^+$ m/z: 708.30; found: 708.10.

Synthesis of compound 1a

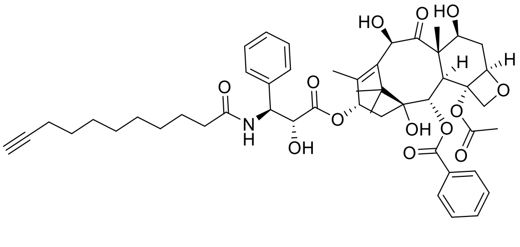

To a solution of 10-undecynoic acid (11.5 mg, 63.4 μmol) in DMF (0.7 mL), triethylamine (26.4 μL, 190 μmol), HBTU (26.4 mg, 69.7 μmol) and the modified docetaxel intermediate (52.5 mg,

69.7 µmol) were added and the reaction mixture was stirred at room temperature for 1.5 h. After complete reaction, 30 mL ethyl acetate was added into the reaction flask, followed by washing with water (30 mL, 2 x) and brine (30 mL). The organic layer was dried over $MgSO_4$ and then evaporated under reduced pressure. The residue was purified by column chromatography to yield the product as a white solid (56%). LC-MS (ESI$^+$): calculated for $C_{49}H_{61}NO_{13}Na$ $[M+Na]^+$ m/z: 894.40; found: 894.15; ^{1}H NMR (400 MHz, MeOD-d_4) δ 8.14 – 8.07 (m, 2H), 7.66 (t, *J* = 7.4 Hz, 1H), 7.56 (t, *J* = 7.6 Hz, 2H), 7.47 – 7.36 (m, 4H), 7.33 – 7.24 (m, 1H), 6.15 (t, *J* = 8.6 Hz, 1H), 5.65 (d, *J* = 7.2 Hz, 1H), 5.46 (d, *J* = 4.5 Hz, 1H), 5.27 (s, 1H), 5.03 – 4.96 (m, 1H), 4.58 (d, *J* = 4.6 Hz, 1H), 4.31 – 4.13 (m, 3H), 3.87 (d, *J* = 7.2 Hz, 1H), 2.51 – 2.40 (m, 1H), 2.34 (s, 3H), 2.31 – 2.19 (m, 3H), 2.19 – 2.11 (m, 3H), 2.02 (dd, *J* = 15.4, 8.8 Hz, 1H), 1.90 (d, *J* = 0.9 Hz, 3H), 1.88 – 1.79 (m, 1H), 1.70 (s, 3H), 1.65 – 1.55 (m, 2H), 1.52 – 1.43 (m, 2H), 1.43 – 1.34 (m, 2H), 1.34 – 1.25 (m, 6H), 1.19 (s, 3H), 1.13 (s, 3H); ^{13}C NMR (101 MHz, MeOD-d_4) δ 209.18, 174.17, 172.51, 169.89, 165.79, 138.26, 137.33, 136.09, 132.63, 129.53, 129.29, 127.78, 127.74, 126.96, 126.52, 84.02, 83.20, 80.46, 77.16, 75.71, 74.55, 73.76, 72.93, 70.77, 70.69, 67.49, 56.98, 54.92, 45.99, 42.64, 35.61, 35.14, 34.92, 28.43, 28.32, 28.15, 27.82, 27.78, 25.21, 25.19, 21.32, 19.77, 17.10, 12.51, 8.56.

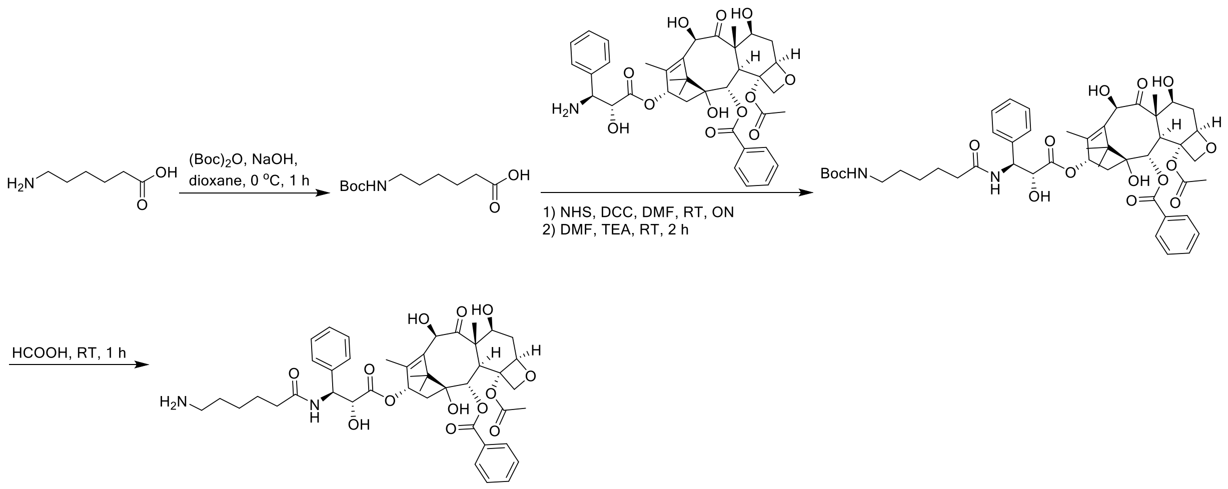

Synthesis of compound S2

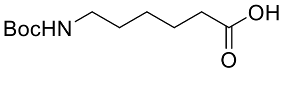

To an ice-cold solution of 6-aminohexanoic (7.87 g, 60 mmol) in 2N NaOH (75 mL, 150 mmol) was added a solution of *tert*-butyl dicarbonate (14.4 g, 66 mmol) in dioxane (75 mL) in portion. Then the reaction mixture was stirred at 0 ℃ for 1 h. After completion of the reaction, the dioxane was removed by evaporation. The aqueous layer was washed with diethyl ether and

acidified by addition of saturated $KHSO_{4\,(aq)}$ to pH 2-3, followed by extraction of EtOAc (80 mL, 3 x). The combined organic layer was washed with brine (80 mL), dried over anhydrous Na_2SO_4, filtered and concentrated to yield the product **S2** as a white foam (13.2 g, 95% yield). LC-MS (ESI$^-$): calculated for $C_{11}H_{20}NO_4^-$ [M-H]$^-$ m/z: 230.14; found: 229.90; ^{1}H NMR (600 MHz, $CDCl_3$) δ 4.60 (s, 1H), 3.13 (d, *J* = 6.0 Hz, 2H), 2.36 (t, *J* = 7.4 Hz, 2H), 1.70 – 1.63 (m, 2H), 1.52 (dd, *J* = 14.8, 7.4 Hz, 2H), 1.45 (s, 9H), 1.41 – 1.35 (m, 2H).

Synthesis of compound S3

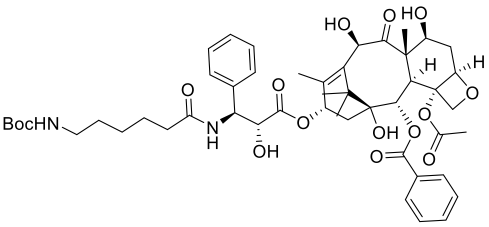

To a solution of **S2** (1.85 g, 8 mmol) in DMF (10 mL) were added DCC (4.95 g, 24 mmol) and *N*-Hydroxysuccinimide (2.76 g, 24 mmol). Then the reaction mixture was stirred at room temperature overnight. After completion of the reaction, the reaction mixture was filtered and the filtrate was evaporated. The crude was resuspended in EtOAc and stored in the freezer for 1 h, followed by filtration, evaporation and drying by high vacuum. The NHS ester product was obtained as a white solid and used for next step directly without further purification. Subsequently, to a solution of **S1** (175 mg, 0.247 mmol) in DMF (4 mL) were added the NHS ester (162 mg, 0.494 mmol) obtained above and triethylamine (103 µL, 0.741 mmol). The reaction mixture was stirred at room temperature for 2 h. After completion of the reaction, the reaction mixture was filtered and water (40 mL) was added into the filtrate, followed by extraction of EtOAc (20 mL, 3 x). The combined organic layer was washed with brine (40 mL), dried over anhydrous Na_2SO_4, filtered and concentrated under reduced pressure. The residue was purified by column chromatography to yield the product **S3** as a white solid (182 mg, 80% yield). LC-MS (ESI$^+$): calculated for $C_{49}H_{64}N_2O_{15}Na^+$ [M+Na]$^+$ m/z: 943.42; found: 943.25; ^{1}H NMR (600 MHz, MeOD-d_4) δ 8.14 – 8.11 (m, 2H), 7.70 – 7.66 (m, 1H), 7.58 (t, *J* = 7.8 Hz, 2H), 7.45 – 7.40 (m, 4H), 7.31 – 7.28 (m, 1H), 6.18 (t, *J* = 8.6 Hz, 1H), 5.67 (d, *J* = 7.2 Hz, 1H), 5.47 (d, *J* = 4.7 Hz, 1H), 5.29 (s, 1H), 5.01 (dd, *J* = 9.6, 1.8 Hz, 1H), 4.60 (s, 1H), 4.24 (dd, *J* = 11.3, 6.6 Hz, 1H), 4.21 (t, *J* = 5.8 Hz, 2H), 3.89 (d, *J* = 7.2 Hz, 1H), 3.00 (t, *J* = 7.1 Hz, 2H), 2.46 (ddd, *J* = 14.5, 9.8, 6.7 Hz, 1H), 2.36 (s, 3H), 2.31 (td, *J* = 7.3, 4.6 Hz, 2H), 2.26 (dd, *J* = 15.3, 9.4 Hz, 1H), 2.06 – 1.99 (m, 1H), 1.92 (s, 3H), 1.85 (ddd, *J* = 13.9, 11.3, 2.2 Hz, 1H), 1.72 (s, 3H), 1.66 – 1.59 (m, 2H), 1.44 (s, 9H), 1.37 – 1.29 (m, 4H), 1.21 (s, 3H), 1.15 (s, 3H). ^{13}C NMR (151 MHz, MeOD-d_4) δ 4209.72, 174.51, 173.04, 170.42, 166.30, 157.13, 138.73, 137.83, 136.58, 133.16, 130.03, 129.79, 128.30, 128.26, 127.49, 127.02, 84.53, 80.95, 78.43, 77.70,

76.22, 75.04, 74.26, 73.47, 71.27, 71.15, 57.49, 55.49, 46.48, 43.15, 39.79, 36.11, 35.52, 35.43, 29.23, 27.41, 26.03, 25.70, 25.35, 21.83, 20.30, 13.03, 9.08.

Synthesis of compound S4

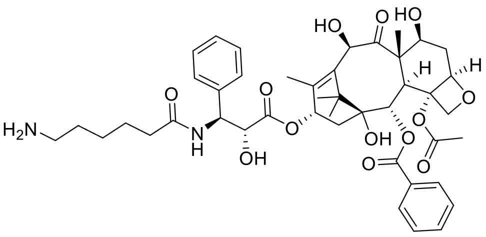

S3 (92 mg, 0.1 mmol) was dissolved in 0.5 mL formic acid and the reaction mixture was stirred for 1 h at room temperature. After completion of the reaction, all solvents were evaporated to yield the intermediate **S4** as a white solid (100% yield), which was used without further purification. LC-MS (ESI$^+$): calculated for $C_{44}H_{57}N_2O_{13}^+$ [M+H]$^+$ m/z: 821.39; found: 821.20.

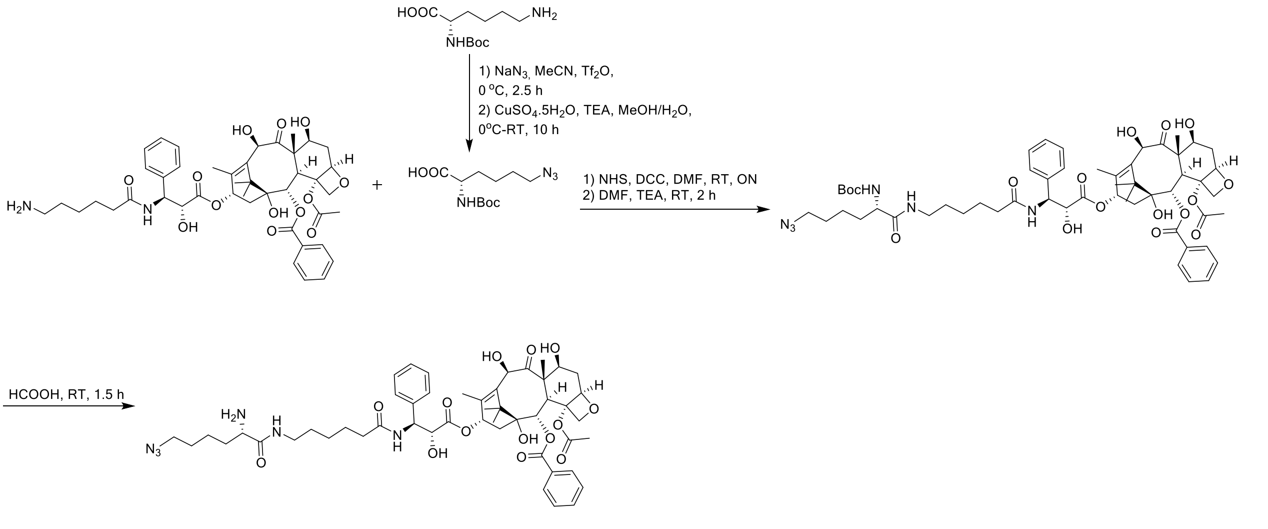

Synthesis of compound S5

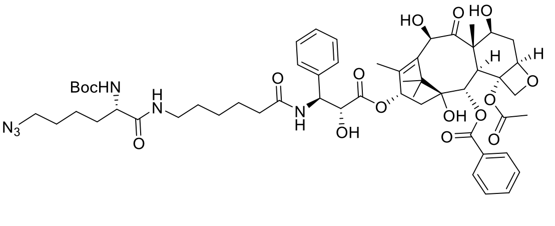

As described in the literature[1], to a suspension of NaN_3 (380 mg, 5.85 mmol) in MeCN (5 mL) was added triflic anhydride (820 µL, 4.87 mmol) in a dropwise manner in an ice bath. Then the reaction mixture was stirred at 0 ℃ for 2.5 h to form triflyl azide. Subsequently, the obtained triflyl azide was diluted with CH_2Cl_2 (35 mL). To a stirred solution of Boc-Lys-OH (1.0 g, 4.06

mmol) in MeOH/water mixture (15 mL/15 mL) were added triethylamine (1.69 mL, 12.18 mmol) and $CuSO_4 \cdot 5H_2O$ (50.7 mg, 0.2 mmol, in 0.5 mL H_2O), followed by addition of the diluted triflyl azide in CH_2Cl_2 through a dropping funnel. Then the reaction mixture was stirred at room temperature for 10 h. After completion of the reaction, the solvent was evaporated and saturated $NaHCO_3$ (40 mL) was added to the residue, followed by extraction of EtOAc (30 mL, 3 x). The aqueous layer was acidified by slow addition of saturated $KHSO_{4(aq)}$ to pH 2-3 and extracted with EtOAc (40 mL, 3 x). The combined organic layer was washed with brine (80 mL), dried over anhydrous Na_2SO_4, filtered and concentrated under reduced pressure to give ***N*^{2}-(*tert*-butoxycarbonyl)-*N*^{6}-diazo-L-lysine** as a yellow oil, which was used to make NHS ester without further purification.

To a solution of ***N*^{2}-(*tert*-butoxycarbonyl)-*N*^{6}-diazo-L-lysine** obtained above in DMF (8 mL) were added DCC (2.26 g, 11 mmol) and *N*-Hydroxysuccinimide (1.26 g, 11 mmol). Then the reaction mixture was stirred at room temperature overnight. After completion of the reaction, the reaction mixture was filtered and the filtrate was evaporated. The crude was resuspended in EtOAc and stored in the freezer for 1 h, followed by filtration, evaporation and drying by high vacuum. The NHS ester product was obtained as a white solid and used for next step directly without further purification. Subsequently, to a solution of **S4** (82 mg, 0.1 mmol) in DMF (2 mL) were added the NHS ester (111 mg, 0.3 mmol) obtained above and triethylamine (41.7 µL, 0.3 mmol). The reaction mixture was stirred at room temperature for 2 h. After completion of the reaction, the reaction mixture was filtered and the filtrate was concentrated under reduced pressure. The residue was purified by column chromatography to yield the product **S5** as a white solid (67 mg, 62% yield). LC-MS (ESI^+): calculated for $C_{55}H_{75}N_6O_{16}^+$ $[M+H]^+$ m/z: 1075.52; found: 1075.65; ^1H NMR (400 MHz, MeOD-d_4) δ 8.17 – 8.09 (m, 2H), 7.70 – 7.65 (m, 1H), 7.58 (t, *J* = 7.6 Hz, 2H), 7.47 – 7.37 (m, 4H), 7.33 – 7.26 (m, 1H), 6.18 (t, *J* = 8.6 Hz, 1H), 5.67 (d, *J* = 7.2 Hz, 1H), 5.52 – 5.44 (m, 1H), 5.29 (s, 1H), 5.17 (s, 4H), 5.01 (dd, *J* = 9.5, 1.7 Hz, 1H), 4.61 (d, *J* = 4.6 Hz, 1H), 4.29 – 4.17 (m, 3H), 3.99 (dd, *J* = 8.1, 5.7 Hz, 1H), 3.89 (d, *J* = 7.1 Hz, 1H), 3.30 (t, *J* = 6.7 Hz, 2H), 3.23 – 3.07 (m, 2H), 2.46 (ddd, *J* = 14.4, 9.7, 6.5 Hz, 1H), 2.36 (s, 3H), 2.34 – 2.20 (m, 3H), 2.04 (dd, *J* = 15.5, 8.8 Hz, 1H), 1.92 (d, *J* = 0.8 Hz, 3H), 1.85 (ddd, *J* = 13.8, 11.4, 2.1 Hz, 1H), 1.72 (s, 3H), 1.69 – 1.56 (m, 5H), 1.55 – 1.48 (m, 2H), 1.45 (s, 9H), 1.44 – 1.27 (m, 5H), 1.21 (s, 3H), 1.15 (s, 3H). ^{13}C NMR (101 MHz, MeOD-d_4) δ 209.72, 174.41, 173.55, 173.05, 170.40, 166.28, 156.39, 138.75, 137.82, 136.59, 133.15, 130.04, 129.81, 128.31, 128.27, 127.49, 127.02, 84.53, 80.95, 79.24, 77.70, 76.23, 75.05, 74.26, 73.47, 71.28, 71.18, 57.49, 55.45, 54.57, 50.88, 46.48, 43.17, 38.77, 36.13, 35.45, 31.67, 28.64, 28.13, 27.36, 26.01, 25.75, 25.24, 22.81, 21.86, 20.33, 18.93, 13.07, 9.11.

Synthesis of compound 1b

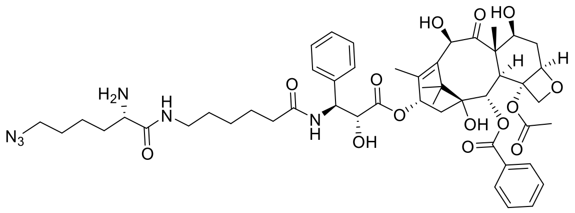

S5 (37 mg, 38 μmol) was dissolved in 0.4 mL formic acid and the reaction mixture was stirred for 1.5 h at room temperature. After completion of the reaction, all solvents were evaporated and the product was dried by high vacuum to yield **1b** as a white solid (100% yield). LC-MS (ESI$^+$): calculated for $C_{50}H_{67}N_6O_{14}^+$ [M+H]$^+$ m/z: 975.47; found: 975.60. ^{1}H NMR (600 MHz, MeOD-d_4) δ 8.13 (d, *J* = 7.3 Hz, 2H), 7.68 (t, *J* = 7.4 Hz, 1H), 7.58 (t, *J* = 7.7 Hz, 2H), 7.45 – 7.40 (m, 4H), 7.30 (t, *J* = 7.0 Hz, 1H), 6.17 (t, *J* = 8.7 Hz, 1H), 5.67 (d, *J* = 7.2 Hz, 1H), 5.48 (d, *J* = 4.6 Hz, 1H), 5.29 (s, 1H), 5.17 (s, 9H), 5.01 (d, *J* = 8.1 Hz, 1H), 4.61 (d, *J* = 4.7 Hz, 1H), 4.27 – 4.18 (m, 3H), 3.89 (d, *J* = 7.1 Hz, 1H), 3.78 (s, 1H), 3.34 (t, *J* = 4.9 Hz, 2H), 3.23 (t, *J* = 6.7 Hz, 2H), 2.46 (ddd, *J* = 14.7, 9.7, 6.6 Hz, 1H), 2.35 (s, 3H), 2.28 (ddd, *J* = 24.8, 15.0, 8.4 Hz, 3H), 2.06 – 2.01 (m, 1H), 1.92 (s, 3H), 1.85 (d, *J* = 6.4 Hz, 1H), 1.72 (s, 3H), 1.68 – 1.59 (m, 5H), 1.57 – 1.51 (m, 2H), 1.46 (dd, *J* = 13.5, 8.8 Hz, 3H), 1.40 – 1.35 (m, 2H), 1.21 (s, 3H), 1.15 (s, 3H). ^{13}C NMR (151 MHz, MeOD-d_4) δ 209.73, 174.38, 173.10, 170.39, 168.71, 166.96, 166.27, 138.73, 137.84, 136.58, 133.17, 130.02, 129.80, 128.31, 128.29, 127.51, 127.01, 84.51, 80.95, 77.71, 76.21, 75.03, 74.27, 73.46, 71.30, 71.19, 57.49, 55.51, 53.05, 50.62, 46.49, 43.15, 39.02, 36.12, 35.45, 35.37, 30.93, 28.54, 28.07, 26.05, 25.74, 25.15, 21.82, 20.31, 18.91, 13.07, 9.09 (little HCOOH left).

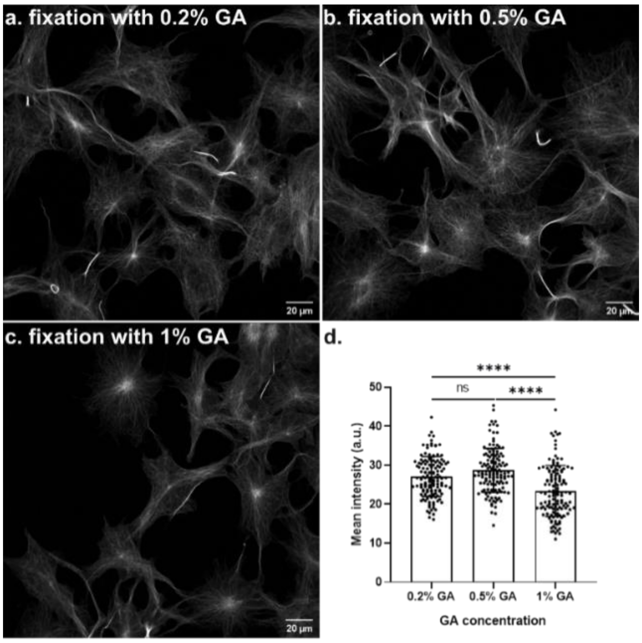

Figure S1 | Comparison of average fluorescence intensity of microtubules obtained after fixation with different concentrations of GA. Specimens labeled with compound **1b** was fixed, permeabilized and click labeled with DBCO-modified Alexa 594. **a**-**c**, Representative images of microtubules fixed with 0.2% (**a**), 0.5% (**b**) and 1% GA (**c**). **d**, Average fluorescence intensity of microtubules achieved after fixation. Bars represent the mean value and error bars represent the standard deviation. Statistical significance was assessed by one-way ANOVA test. **** $p < 0.0001$, ns (non-significant) = 0.0613. From left to right, mean values are 27.10 ± 5.11 (mean ± standard deviation, n =132 from three independent samples), 28.72 ± 5.65, 23.39 ± 6.55, respectively. Representative images from n=3 independent samples.

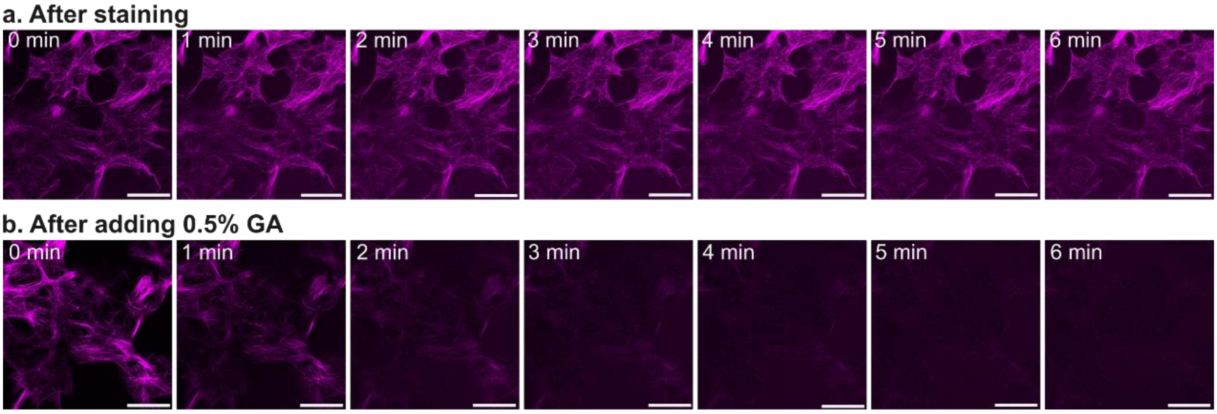

Figure S2 | Evaluation of the fixability of SiR-tubulin. **a**, Time-lapse recording of fluorescent signal of SiR-tubulin-labeled microtubules in living COS-7 cells. **b**, Time-lapse recording of fluorescent signal of microtubules stained with SiR-tubulin after fixing cells with 0.5% GA. Representative images were from two independent samples. Scale bars, 30 µm.

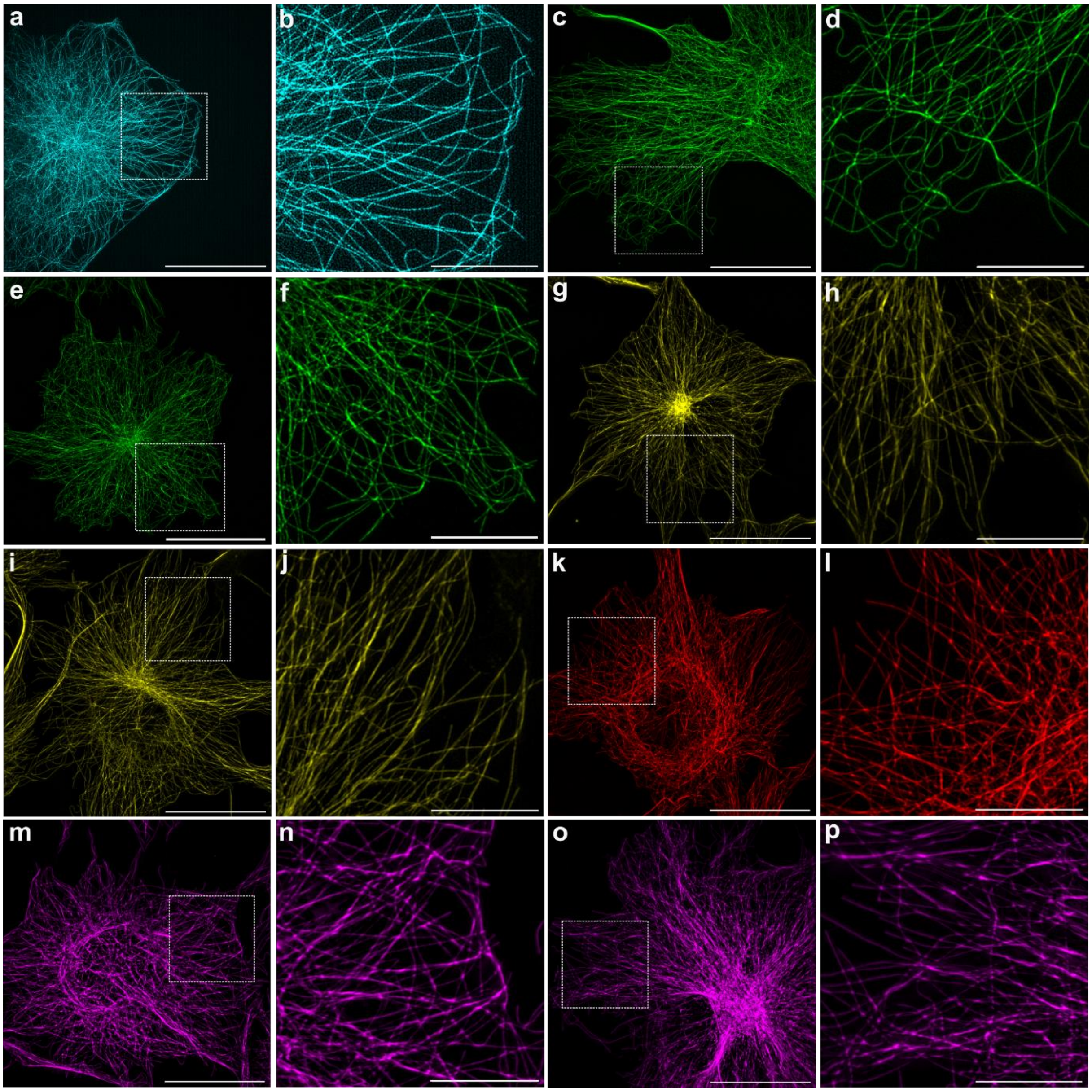

Figure S3 | SIM image of microtubules in COS-7 cells stained with compound 1b and click-labeled with various organic dyes. Cells fed with compound **1b** were fixed, permeabilized and stained with different DBCO-modified organic dyes. **a**,**b**, SIM image of microtubules with Pacific blue. **c**,**d**, SIM image of microtubules with Bodipy FL. **e**,**f**, SIM image of microtubules with Alexa Fluor 488. **g**,**h**, SIM image of microtubules with Rhodamine B. **i**.**j**. SIM image of microtubules with Alexa Fluor 568. **k**,**l**, SIM image of microtubules with Alexa Fluor 594. **m**,**n**, SIM image of microtubules with Alexa Fluor 647. **o**,**p**, SIM image of microtubules with ATTO 643. Representative images were from three independent samples. Scale bars, 30 μm (a,c,e,g,i,k,m,o), 10 μm (b,d,f,h,j,l,n,p).

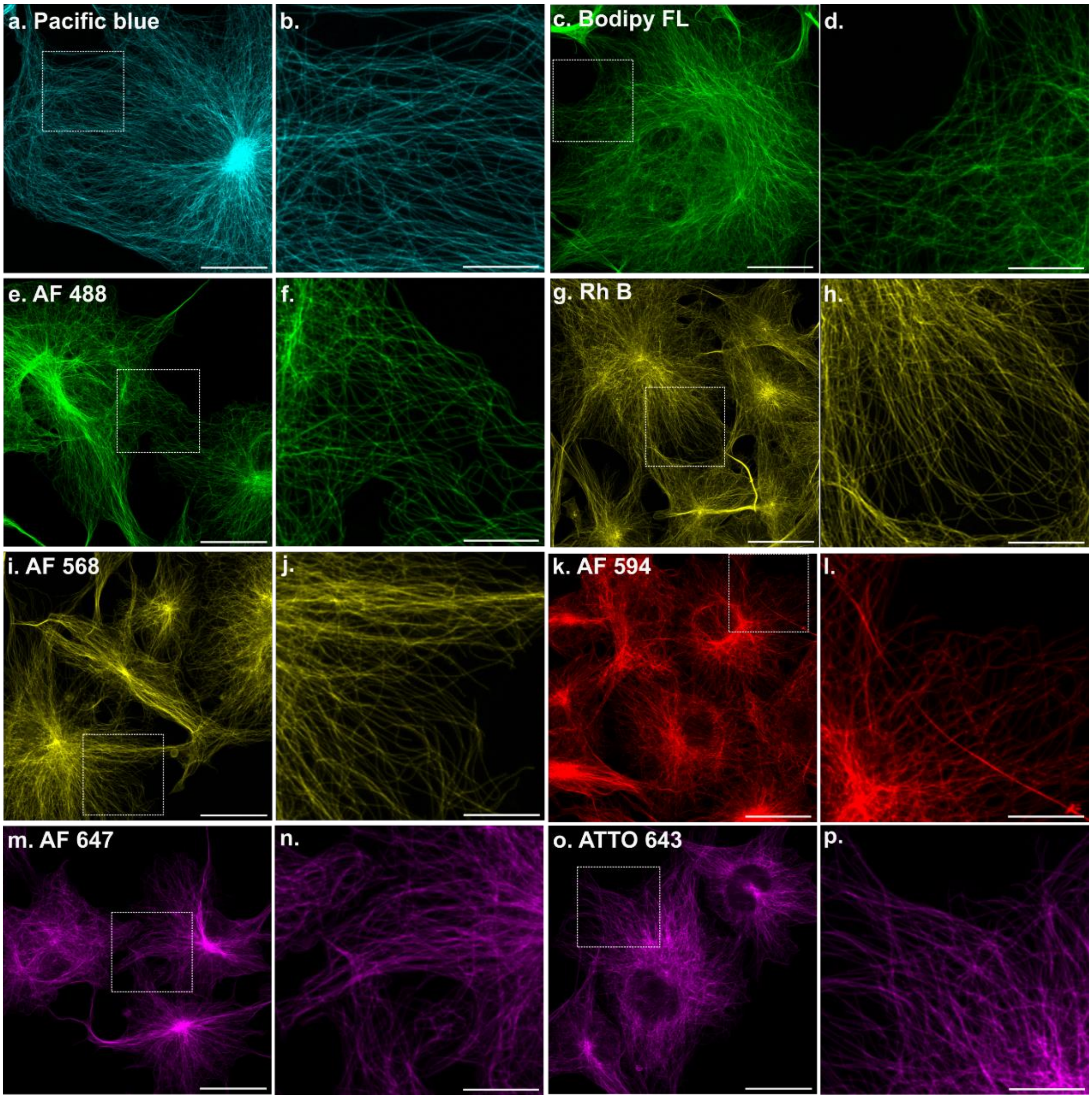

Figure S4 | Airyscan microscopy images of microtubules in COS-7 cells with probe **1b** and various organic dyes. **a**,**b**, Airyscan SR image of microtubules with Pacific blue. **c**,**d**, Airyscan SR image of microtubules with Bodipy FL. **e**,**f**, Airyscan SR image of microtubules with AF 488. **g**,**h**, Airyscan SR image of microtubules with Rhodamine B. **i**,**j**, Airyscan SR image of microtubules with AF 568. **k,l**, Airyscan SR image of microtubules with AF 594. **m**,**n**, Airyscan SR image of microtubules with AF 647. **o**,**p**, Airyscan SR image of microtubules with ATTO 643. Representative images were from three independent samples. Scale bars, 30 μm (**a**,**c**,**e**,**g**,**i**,**k**,**m**,**o**), 10 μm (**b**,**d**,**f**,**h**,**j**,**l**,**n**,**p**).

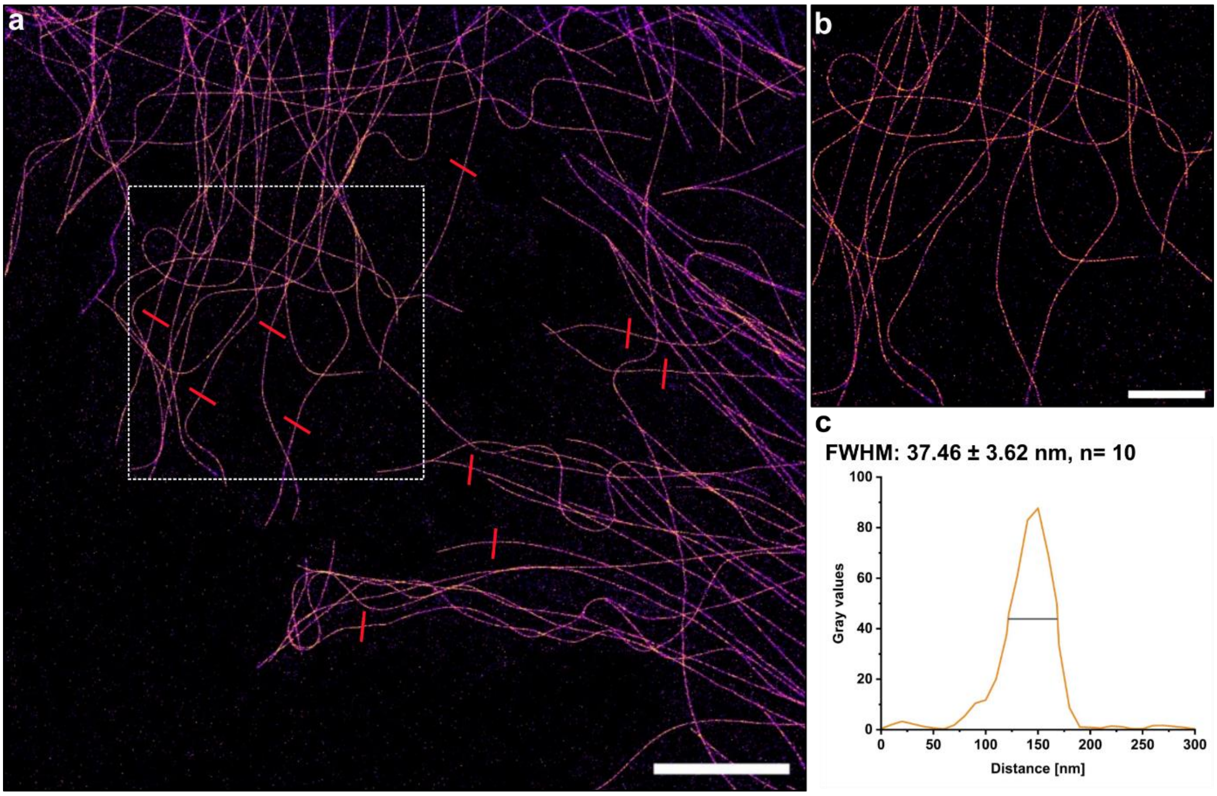

Figure S5 | *d*STORM image of microtubules in COS-7 cells using compound 1b. After fixation and permeabilization, compound **1b**-labeled microtubules were stained with AF647-DBCO. **a**,**b**, *d*STORM image of microtubules stained with AF647 (**a**) and the magnified view (**b**). **c**, Analysis of cross-sectional intensity profile of 10 microtubule profiles shown in **a** (red lines). Representative images were taken from two independent samples. Scale bars, 5 µm (a), 2 µm (b).

Table S1. Microtubule staining in different cell lines with compound **1b**.

No.	Cell lines	Organism	Providers	Staining
1	COS-7	Cercopithecus aethiops	CLS	+
2	U2OS	Human Osteosarcoma	CLS	+
3	Hela	Homo sapiens	CLS	+
4	Hela-GFP (HK-2xZen-mEGFP-Nup107)	Homo sapiens	CLS	+
5	Macrophase	Mus musculus	ATCC	+
6	HEK 293T	Homo sapiens	DSMZ	+
7	Raji	Homo sapiens	ATCC	-
8	Primary hippocampal neurons (E18 C57BL/6)	Mus musculus	Self-prepared	+

Note: "+" Specific staining of microtubules in the cell population; "-" No specific staining of microtubules in the cell population; CLS: Cell Lines Service GmbH; DSMZ: Deutsche Sammlung von Mikroorganismen und Zellkulturen GmbH; ATCC: American Type Culture Collection.

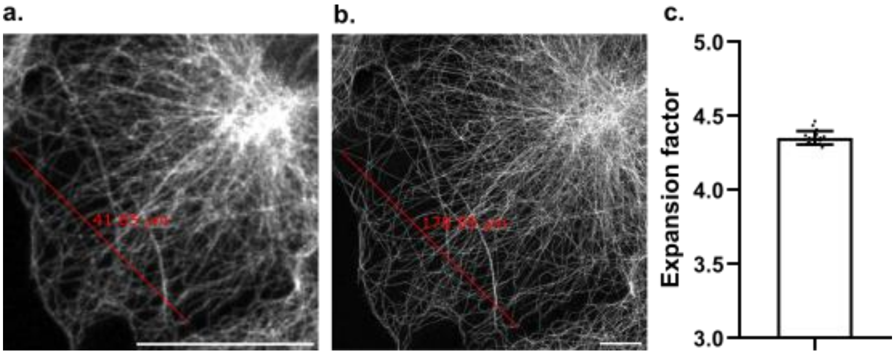

Figure S6 | Expansion factor determination using the original ExM protocol. **a**, Representative image of microtubules before expansion. **b**, Post-expansion image of the same microtubules. **c**, Analysis of expansion factor obtained after 4x ExM, yielding an expansion factor of 4.3 ± 0.046 (mean ± standard deviation, n= 18). Scale bars, 30 µm.

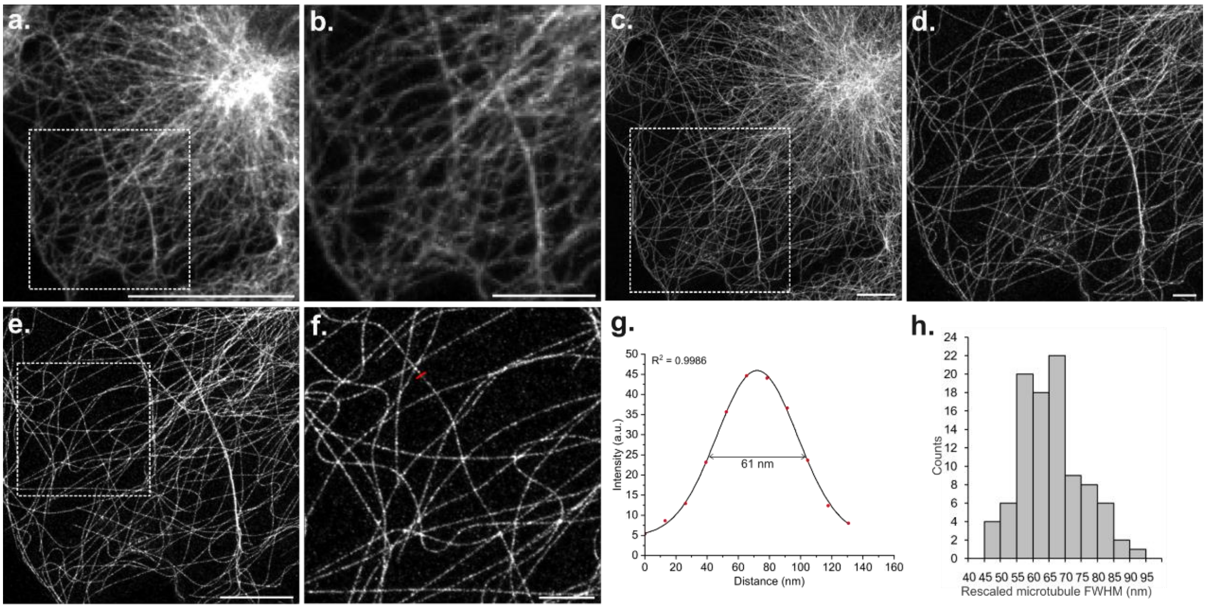

Figure S7 | Microtubules visualized by 4x-ExM-Airyscan SR. **a**-**f**, Comparison of pre- and post-expansion images of microtubules (CF 568) in cultured COS-7 cells in 4x ExM. **a**, Pre-expansion confocal fluorescence image of microtubules in 4x ExM. **b**, Magnified views of the boxed region in panel **a**. **c**, Post-expansion confocal fluorescence image of microtubules in the same cell. **d**, Magnified views of the boxed region in panel **c**. **e**, Airyscan SR image of the same area in panel **d**. **f**, Zoom-in views of the highlighted region in panel **e**. **g**, A representative cross-sectional intensity profile of microtubules (red dots) with Gaussian fitting (solid line). **h**, Distribution of Gaussian-fitted full width at half-maximum (FWHM) of 96 microtubule intensity profiles, yielding a resolution of 65.68 ± 9.54 nm (mean ± s.d., n= 96). Representative images were from two independent samples. Scale bars: 30 µm (**a**,**c**,**e**), and 10 µm (**b**,**d**,**f**).

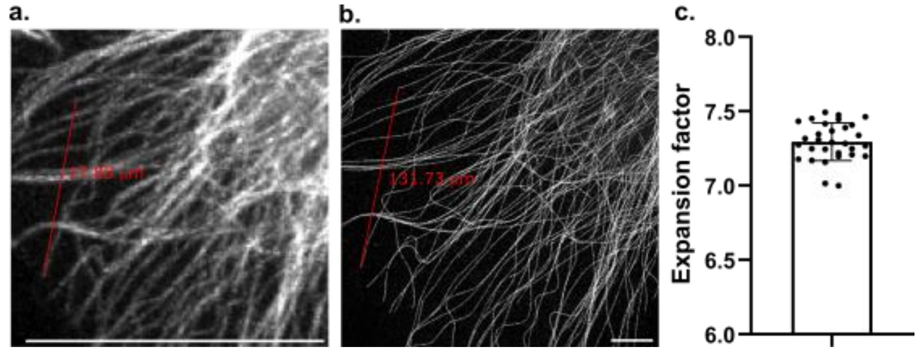

Figure S8 | Expansion factor calculation for microtubules expanded using the TREx protocol. **a**, Representative image of microtubules before expansion. **b**, Post-expansion image of the same microtubules. **c**, Analysis of expansion factor obtained after TREx, yielding an expansion factor of 7.3 ± 0.13 (mean ± standard deviation, n= 30). Scale bar, 30 µm.

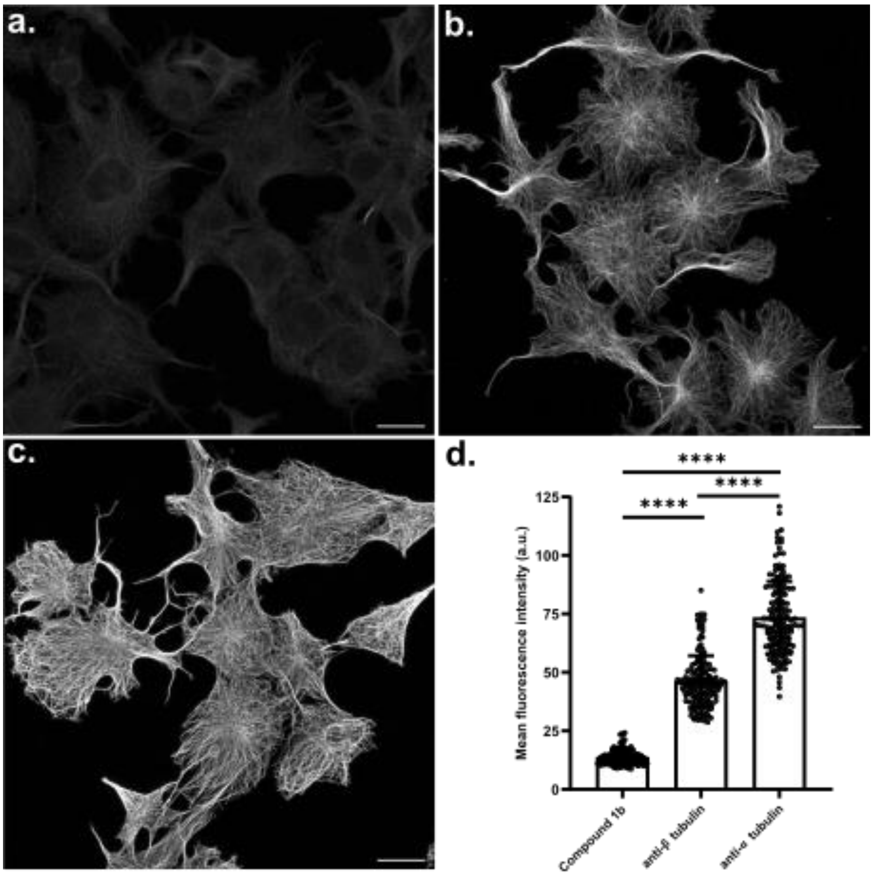

Figure S9 | Comparison of fluorescence signal of astral microtubules stained with different strategies. **a**, Representative image of microtubules stained with compound **1b**. COS-7 cells stained with compound **1b** were fixed and permeabilized, followed by click-reaction with DBCO-biotin and staining with CF568-modified streptavidin. **b**, Representative image of microtubules stained with anti-β tubulin antibodies. COS-7 cells were fixed and permeabilized, followed by staining with primary anti-β tubulin antibodies (Mouse) and CF-568-conjugated secondary antibodies (Goat Anti-Mouse IgG). **c**, Representative image of microtubules stained with anti-α tubulin antibodies. COS-7 cells were fixed and permeabilized, followed by staining with primary anti-α tubulin antibodies (Rabbit) and CF-568-conjugated secondary antibodies (Goat Anti-Rabbit F(ab)$_2$ IgG). **d**, Average fluorescence intensity of microtubules stained with compound **1b** and anti-tubulin antibodies. Bars represent the mean value and error bars represent the standard deviation. Statistical significance was assessed by one-way ANOVA test. **** $p < 0.0001$. From left to right, mean values are 13.58 ± 3.07 (mean ± standard deviation), 46.14 ± 11.02, and 73.33 ± 15.54 (n = 167 cells were measured from three independent samples), respectively. Scale bars, 30 µm.

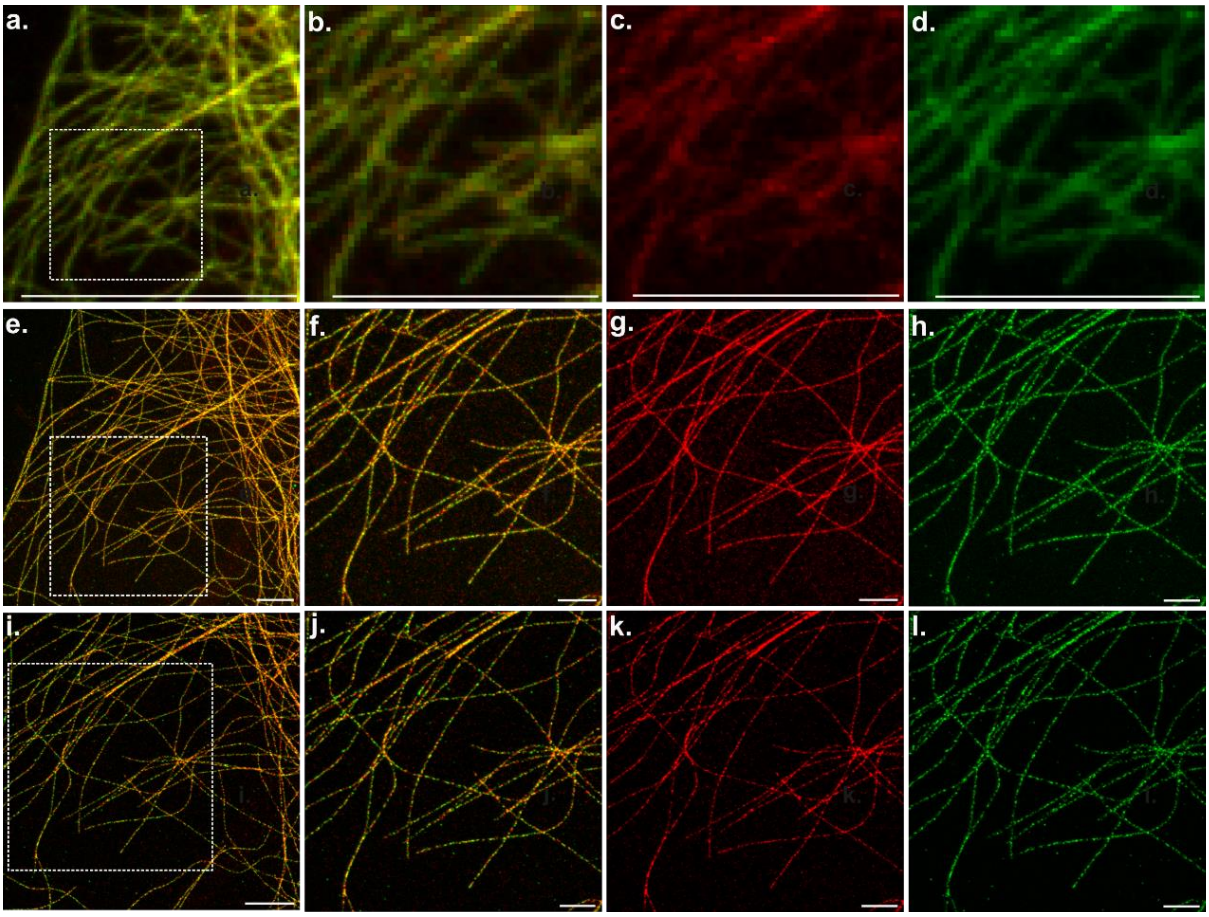

Figure S10 | Multi-color images of microtubules stained with compound **1b** and anti-β tubulin antibodies in TREx. **a**, Pre-expansion confocal fluorescence image of microtubules visualized with compound **1b** (CF 568, red) or immunostained β-tubulin (AF 488, green). **b**, Magnified views of the boxed region in panel **a**. **c**,**d**, Red and green channel in panel **b**. **e**, Post-expansion confocal fluorescence images of microtubules obtained after TREx in the same cell. **f**, Magnified views of the boxed region in panel **e**. **g**,**h**, Red and green channel in panel **f**. **i**, Airyscan SR image of the same cell after TREx. **j**, Magnified views of the boxed region in panel **i**. **k**,**l**, Red and green channel in panel **j**. Representative images were from three independent samples. Scale bars, 20 µm (**a**,**e**,**i**) and 10 µm (**b**-**d**,**f**-**h**,**j**-**l**).

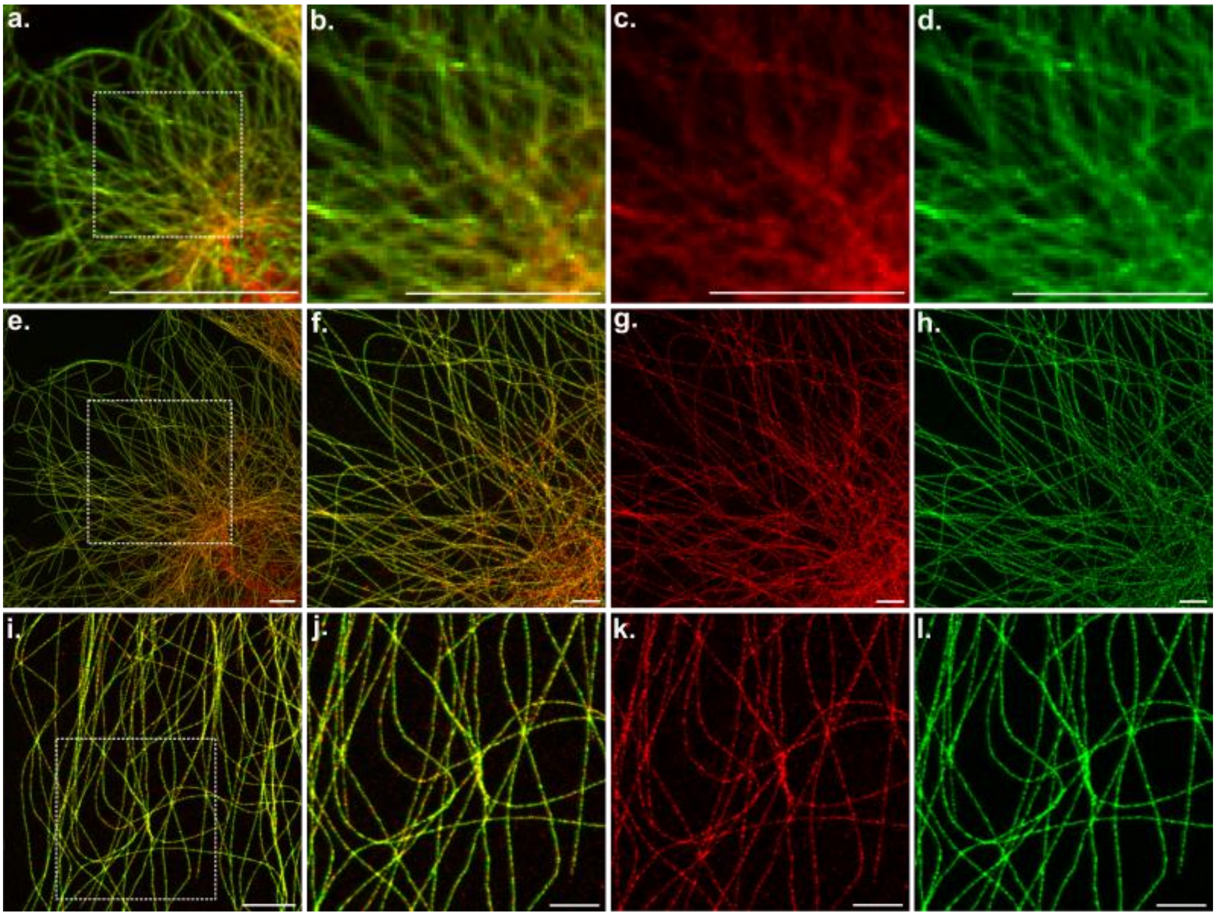

Figure S11 | Multi-color images of microtubules stained with compound **1b** and anti- α tubulin antibodies in TREx. **a**, Pre-expansion confocal fluorescence image of microtubules visualized with compound **1b** (CF 568, red) or immunostained α-tubulin (AF 488, green). **b**, Magnified views of the boxed region in panel **a**. **c**,**d** Red and green channel in panel **b**. **e**, Post-expansion confocal fluorescence image of microtubules obtained after TREx in the same cell. **f**, Magnified views of the boxed region in panel **e**. **g**,**h** Red and green channel in panel **f**. **i**, Airyscan SR image of microtubules in different cells after TREx. **j** Magnified views of the boxed region in panel **i**. **k**,**l**, Red and green channel in panel **j**. Representative images were from three independent samples. Scale bars: 20 µm (**a**,**e**,**i**), and 10 µm (**b**-**d**,**f**-**h**,j-**l**).

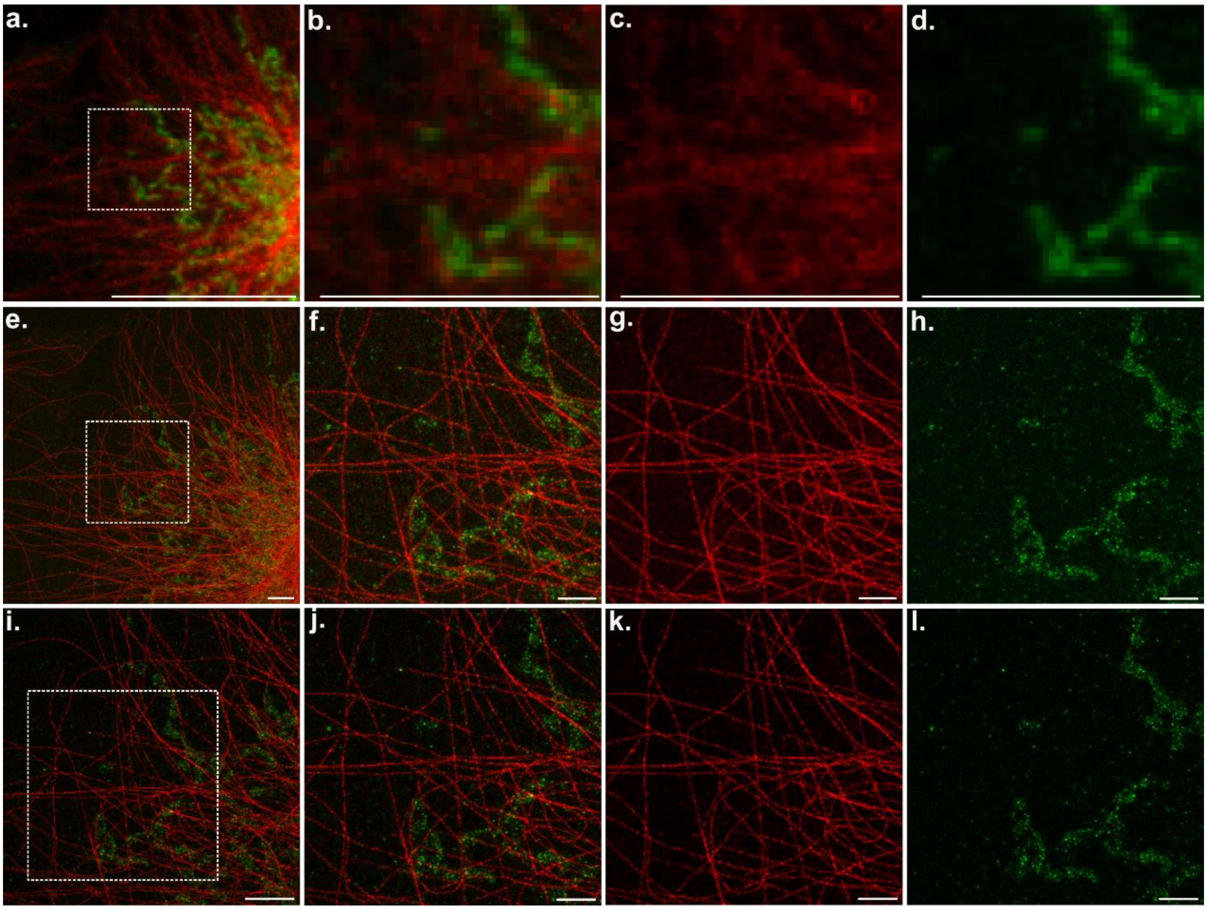

Figure S12 | Multi-color images of compound **1b**-labeled microtubules and immunostained mitochondria in TREx. **a**, Pre-expansion confocal fluorescence image of microtubules visualized with compound **1b** (CF 568, red) or immunostained mitochondria (AF 488, TOMM20). **b**, Magnified views of the boxed region in panel **a**. **c**,**d**, Red and green channel in panel **b**. **e**, Post-expansion confocal fluorescence image of microtubules obtained after TREx in the same cell. **f**, Magnified views of the boxed region in panel **e**. **g**,**h** Red and green channel in panel **f**. **i**, Airyscan SR image of the same cell after TREx. **j**, Magnified views of the boxed region in panel **i**. **k**,**l**, Red and green channel in panel **j**. Representative images were from three independent samples Scale bars, 20 µm (**a**,**e**,**i**), and 10 µm (**b**-**d**,**f**-**h**,**j**-**l**).

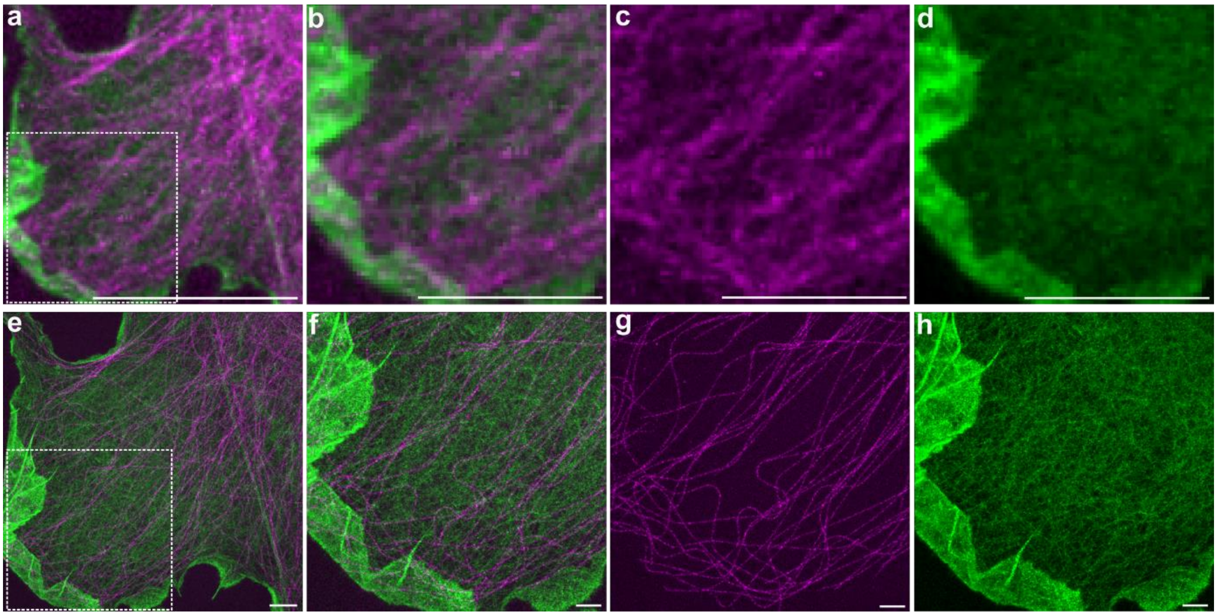

Figure S13 | Multi-color images of compound **1b**-labeled microtubules and actin filaments stained with phalloidin in COS-7 cells using TREx. **a**, Pre-expansion confocal fluorescence image of microtubules visualized with compound **1b** (ATTO 643, magenta) or actin filaments (‘Actin ExM’, 561, green). **b**, Magnified views of the boxed region in panel **a**. **c**,**d** Magenta and green channel in **b**. **e**, Post-expansion confocal fluorescence image of microtubules obtained after TREx in the same cell. **f**, Magnified views of the boxed region in **e**. **g**.**h**, Magenta and green channel in **f**. Representative images were from three independent samples. Scale bars, 20 µm (a,e), 10 µm (b-d,f-h).

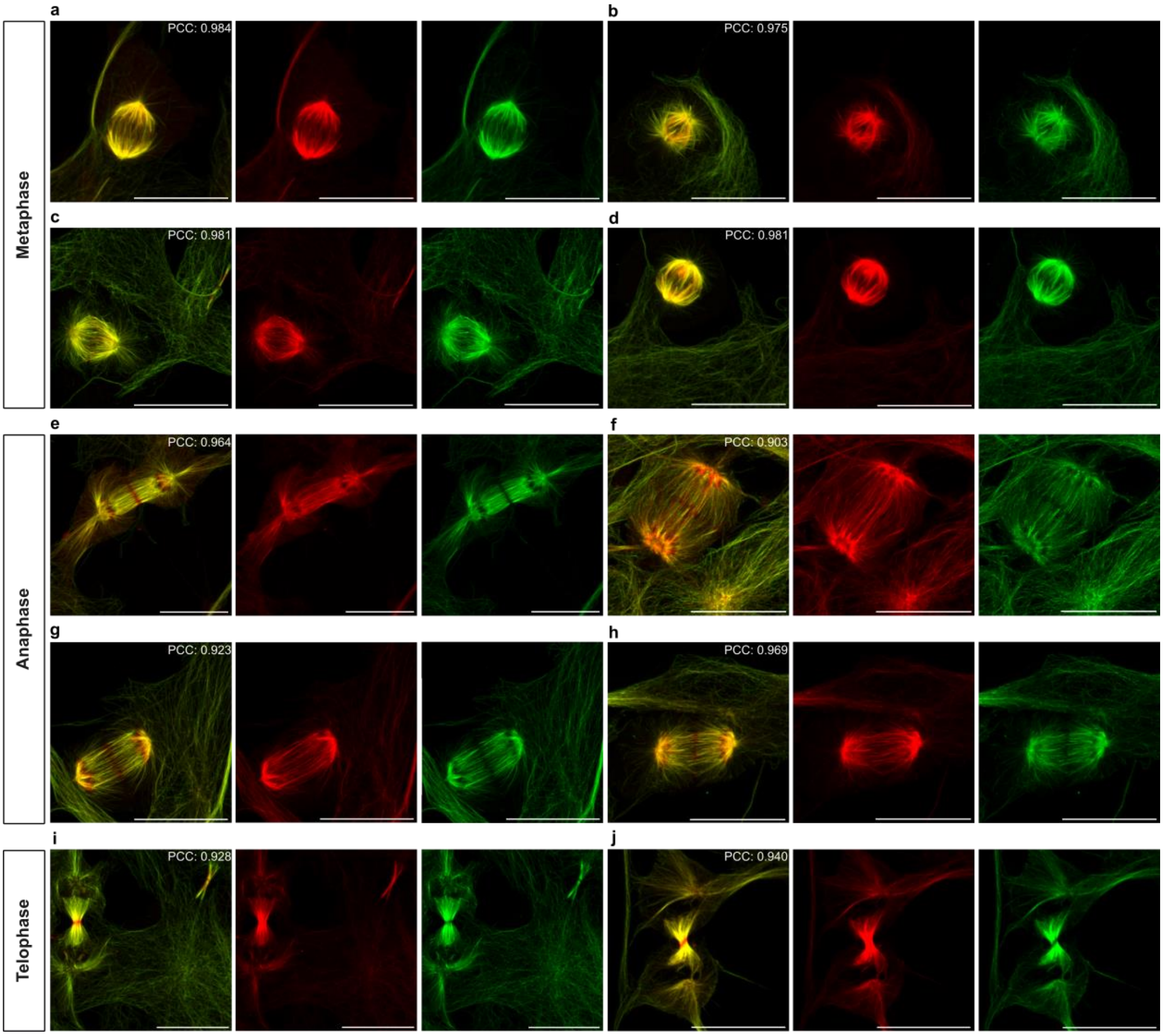

Figure S14 | Comparison of microtubules labeled in COS-7 cells with compound **1b** and anti-β tubulin antibodies at different cell cycle stages by airyscan microscopy. **a**-**d**, Two-color images of microtubules stained with compound **1b** (CF 568, red) and anti-β tubulin antibodies (AF 488, green) at metaphase. **e-h**, Two-color images of microtubules stained with compound **1b** (CF 568, red) and anti-β tubulin antibodies (AF 488, green) at anaphase. **i**,**j**, Two-color images of microtubules stained with compound **1b** (CF 568, red) and anti-β tubulin antibodies (AF 488, green) at telophase. Pearson's correlation coefficient (PCC) was applied to quantify the colocalization coefficients of mitotic microtubules stained with compound **1b** or anti-β tubulin antibodies and shown in the merged channels. Representative images were from n=3 independent samples. Scale bars, 20 µm.

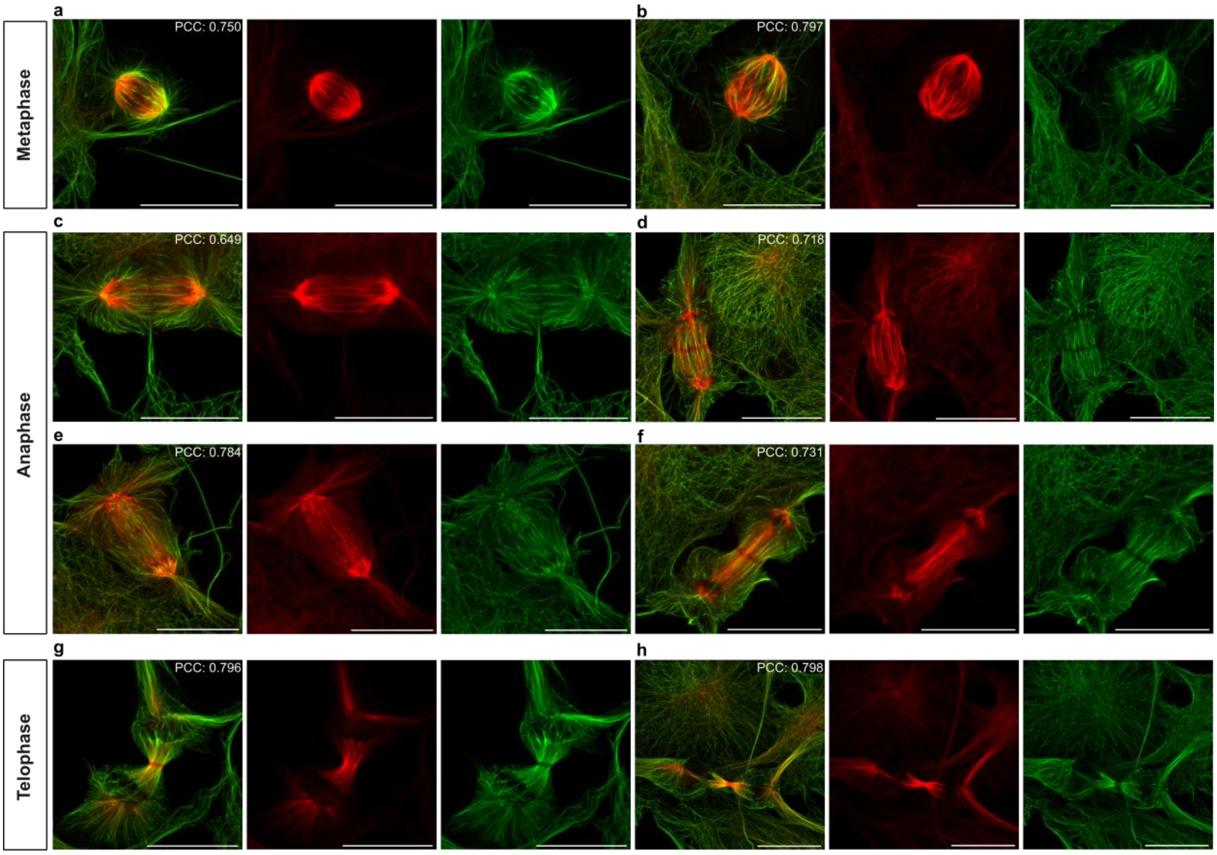

Figure S15 | Comparison of microtubules labeled in COS-7 cells with compound **1b** and anti-α tubulin antibodies at different cell cycle stages by airyscan microscopy. **a**,**b**, Two-color images of microtubules stained with compound **1b** (CF 568, red) and anti- α tubulin antibodies (AF 488, green) at metaphase. **c**-**f**, Two-color images of microtubules stained with compound **1b** (CF 568, red) and anti- α tubulin antibodies (AF 488, green) at anaphase. **g**,**h**, Two-color images of microtubules stained with compound **1b** (CF 568, red) and anti-α tubulin antibodies (AF 488, green) at telophase. Pearson's correlation coefficient (PCC) was applied to quantify the colocalization coefficients of mitotic microtubules stained with compound **1b** or anti-α tubulin antibodies and shown in the merged channels. Representative images were from n=2 independent samples. Scale bars, 20 µm.

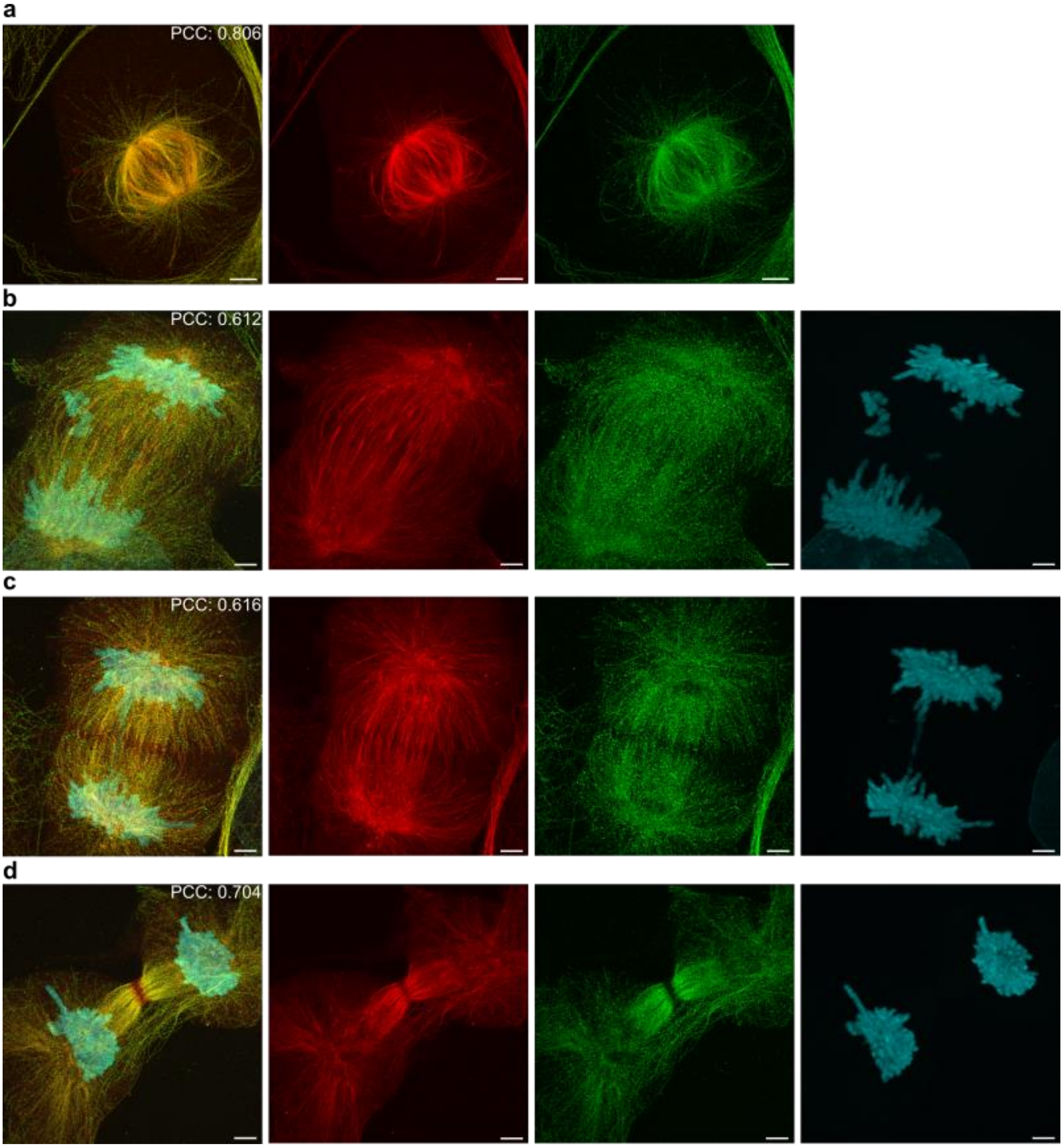

Figure S16 | Comparison of TREx-expanded microtubules in mitotic COS-7 cells using compound **1b** and antibodies to β tubulin. Microtubules stained with compound **1b** (CF568, red) or with anti-β tubulin antibodies (AF 488, green) and nuclei stained with DAPI (cyan) are shown. **a**, Two-color images of microtubules in metaphase cells. **b**,**c**, Three-color images of microtubules and nuclei in anaphase cells. **d**, Three-color images of microtubules and nuclei in telophase cells. Pearson's correlation coefficient (PCC) was applied to quantify the colocalization coefficients of mitotic microtubules stained with compound **1b** or anti-β tubulin antibodies and shown in the merged channels. Representative images were from three independent samples. Scale bars, 20 µm.

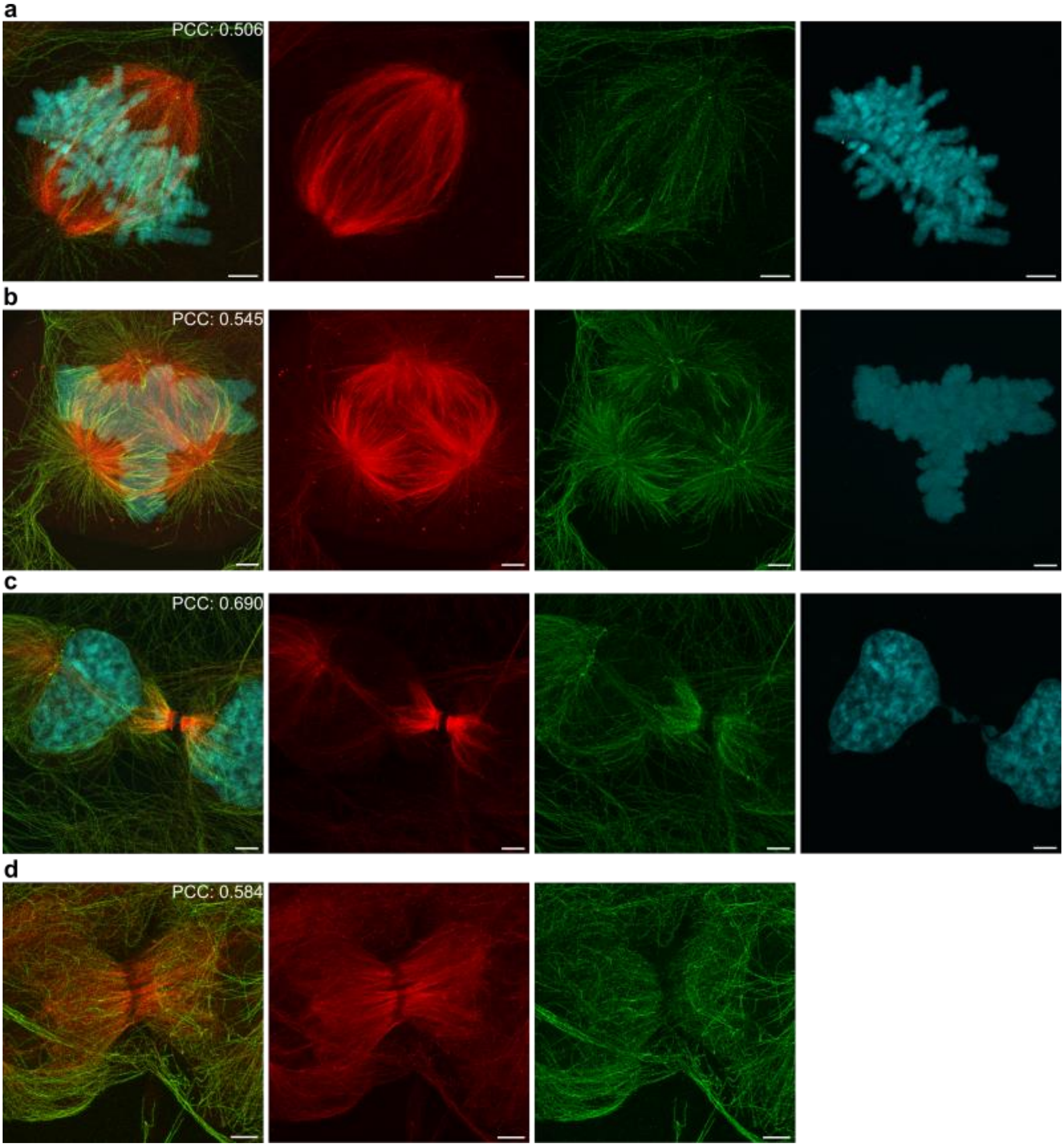

Figure S17 | Comparison of TREx-expanded microtubules in mitotic COS-7 cells using compound **1b** and antibodies to α-tubulin. Microtubules stained with compound **1b** (CF568, red) or with anti-α tubulin antibodies (AF 488, green) and nuclei stained with DAPI (cyan) are shown. **a**,**b** Three-color images of microtubules and nuclei in metaphase cells. **c**,**d** Three-color images of microtubules and nuclei in telophase cells. Pearson's correlation coefficient (PCC) was applied to quantify the colocalization coefficients of mitotic microtubules stained with compound **1b** or anti-α tubulin antibodies and shown in the merged channels. Representative images were from four independent samples. Scale bars, 20 μm.

^{1}H and ^{13}C NMR spectra

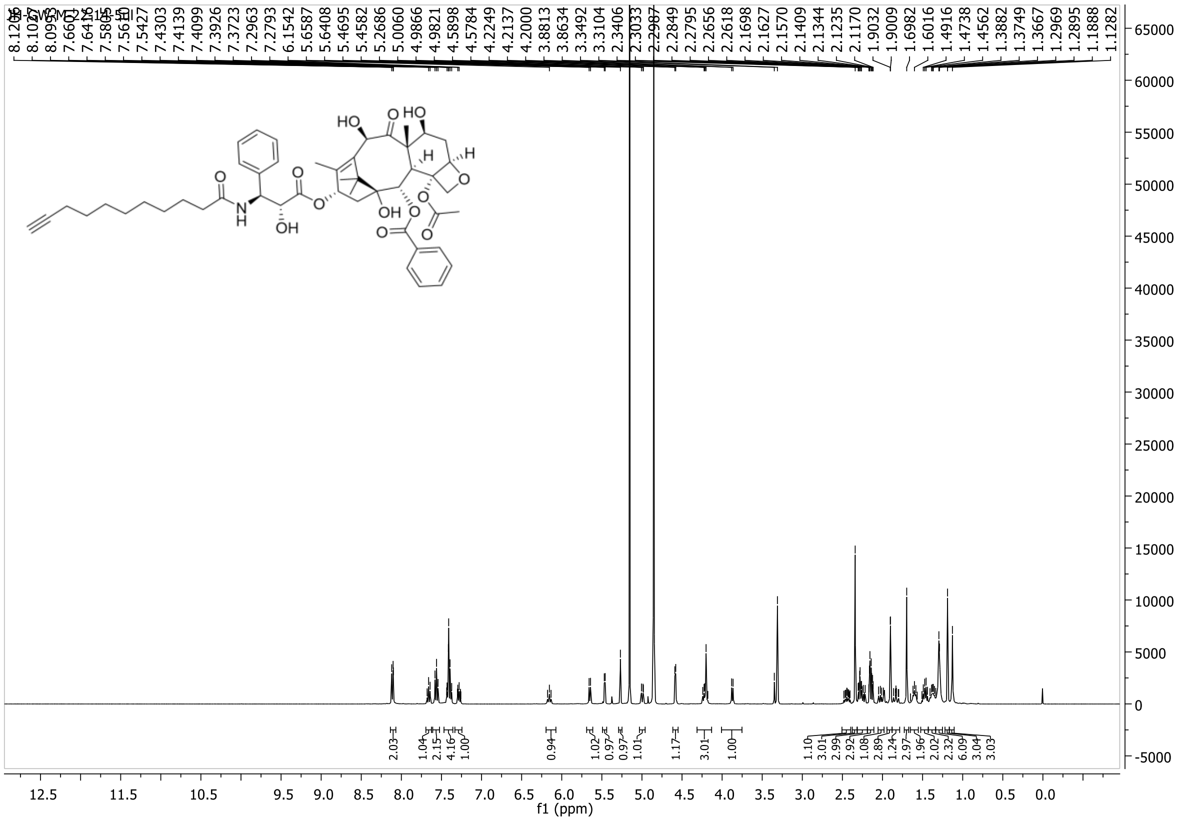

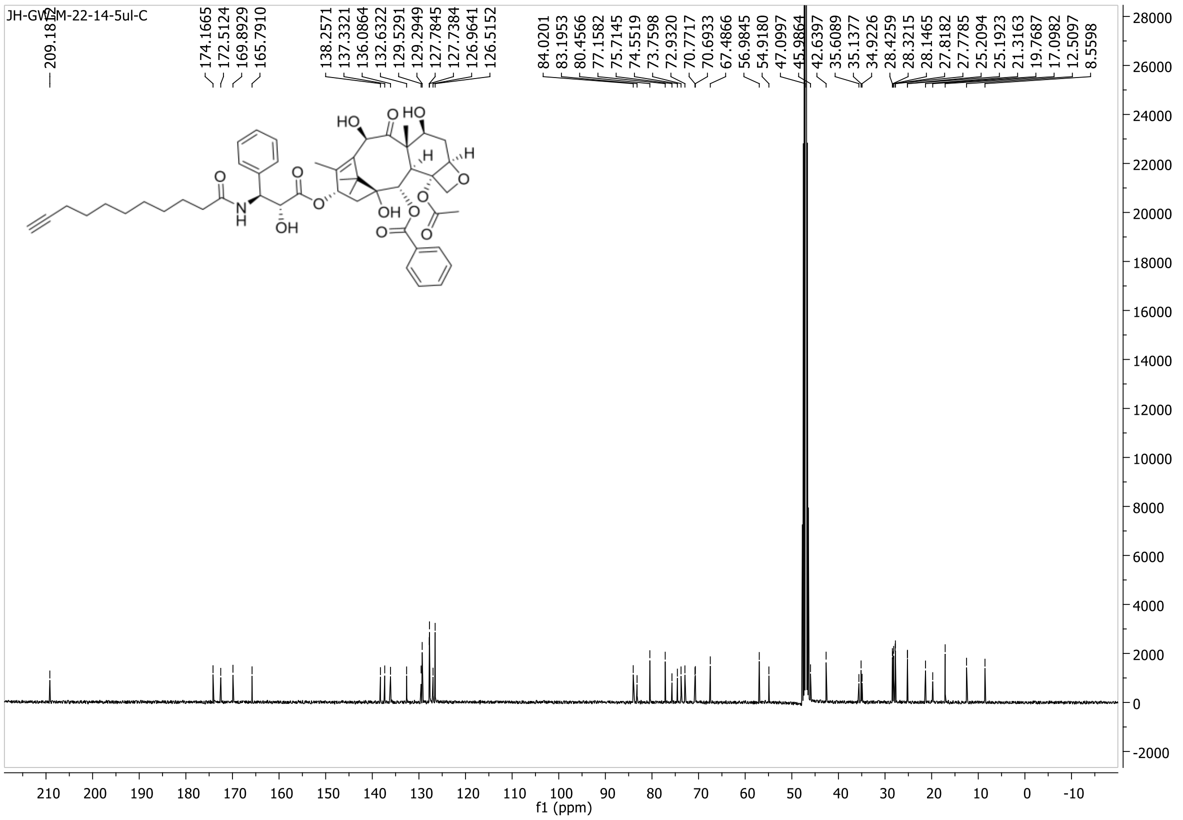

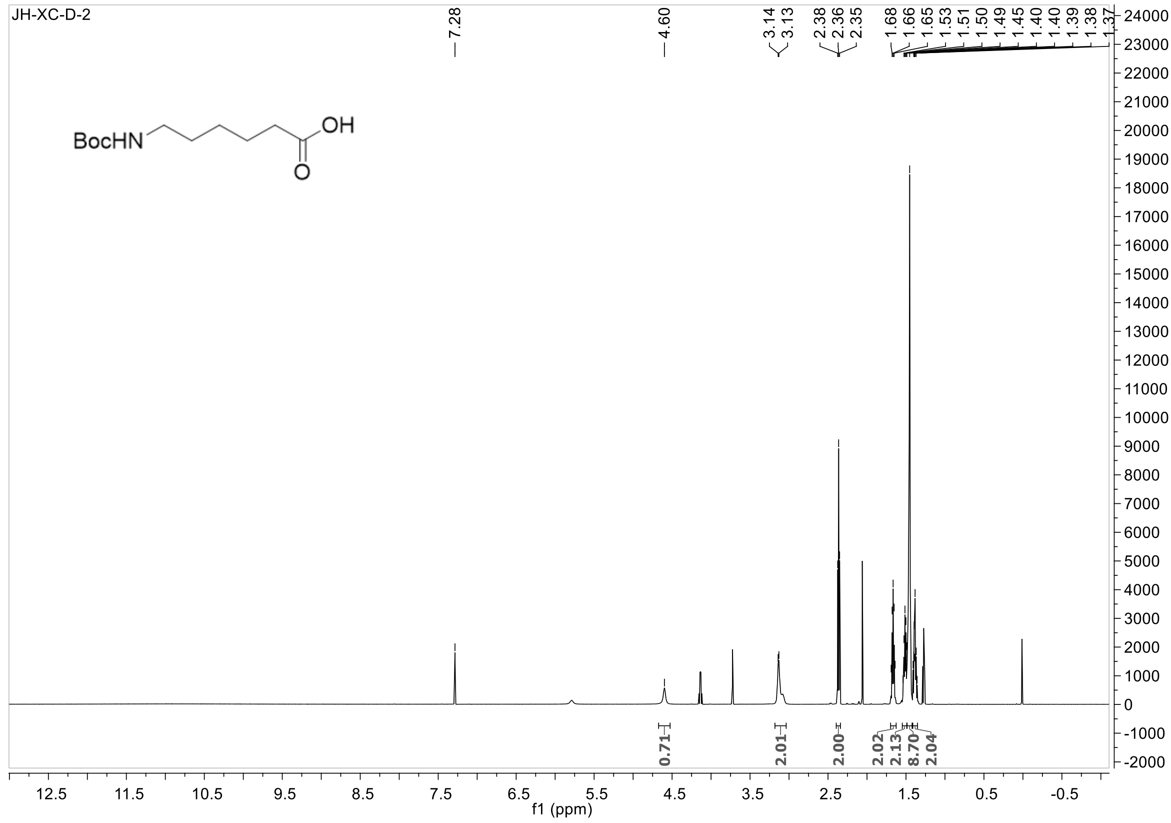
JH-XC-D-2
BocHN
OH
O
7.28
4.60
3.14
3.13
2.38
2.36
2.35
1.68
1.66
1.65
1.53
1.51
1.50
1.49
1.45
1.40
1.39
1.38
1.37
0.71
2.01
2.00
2.02
2.13
8.70
2.04
f1 (ppm)

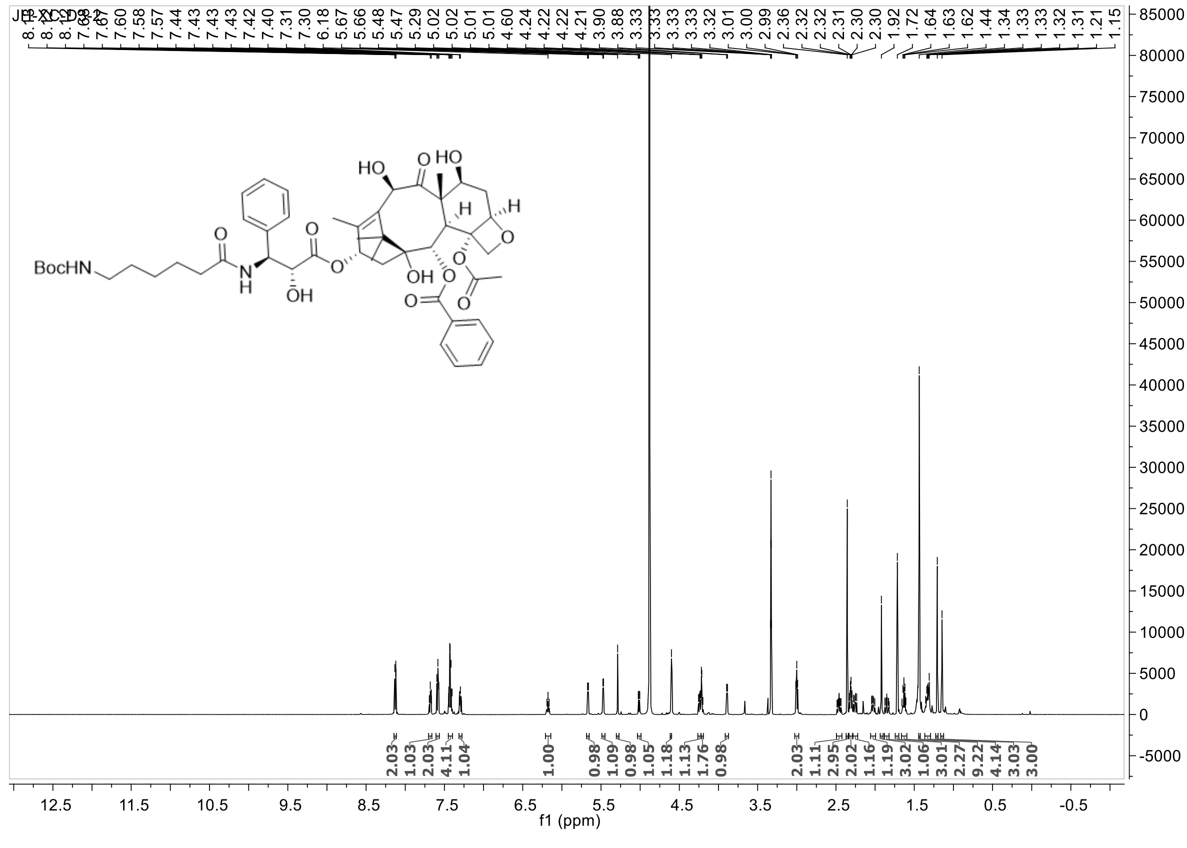
BocHN
N
H
OH
O
HO
OH
H
f1 (ppm)

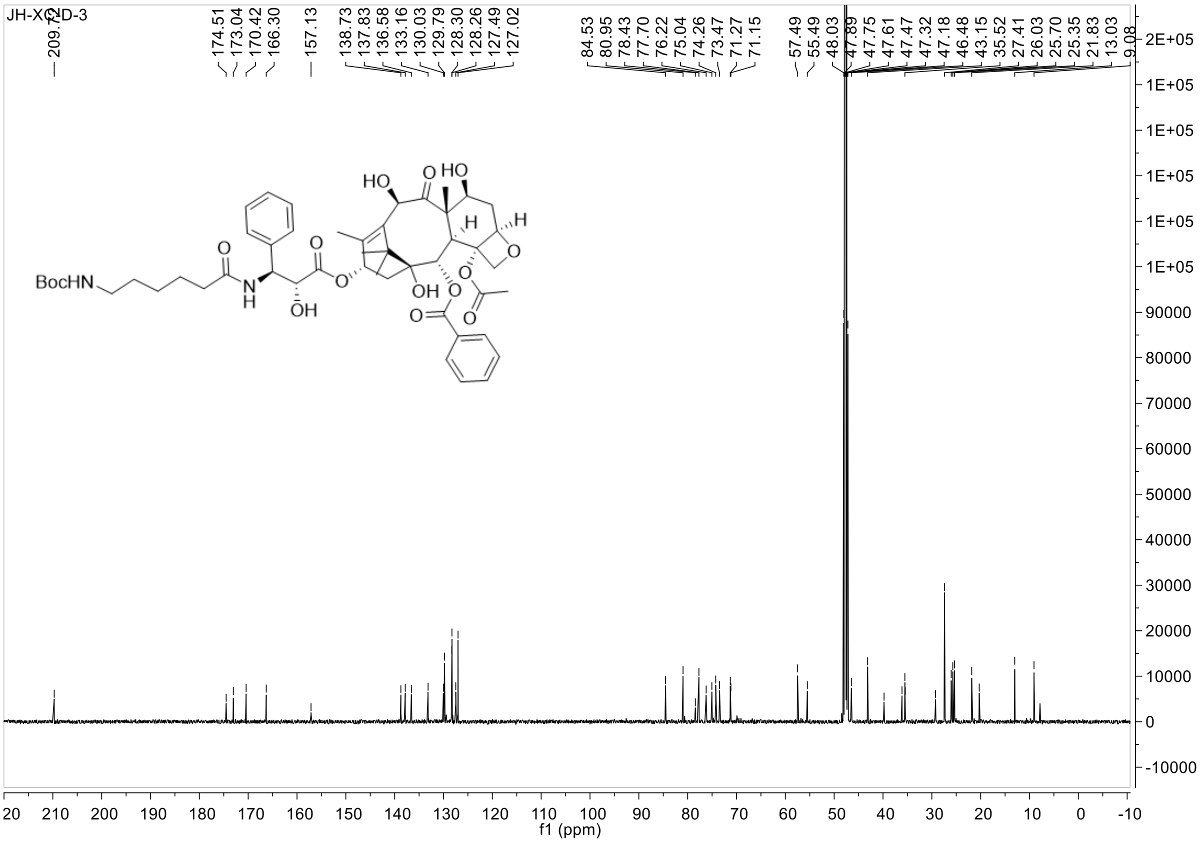
JH-XCND-3
209.72
174.51
173.04
170.42
166.30
157.13
138.73
137.83
136.58
133.16
130.03
129.79
128.30
128.26
127.49
127.02
84.53
80.95
78.43
77.70
76.22
75.04
74.26
73.47
71.27
71.15
57.49
55.49
48.03
47.89
47.75
47.61
47.47
47.32
47.18
46.48
43.15
35.52
27.41
26.03
25.70
25.35
21.83
13.03
9.08
BocHN
N
H
O
OH
HO
H
f1 (ppm)

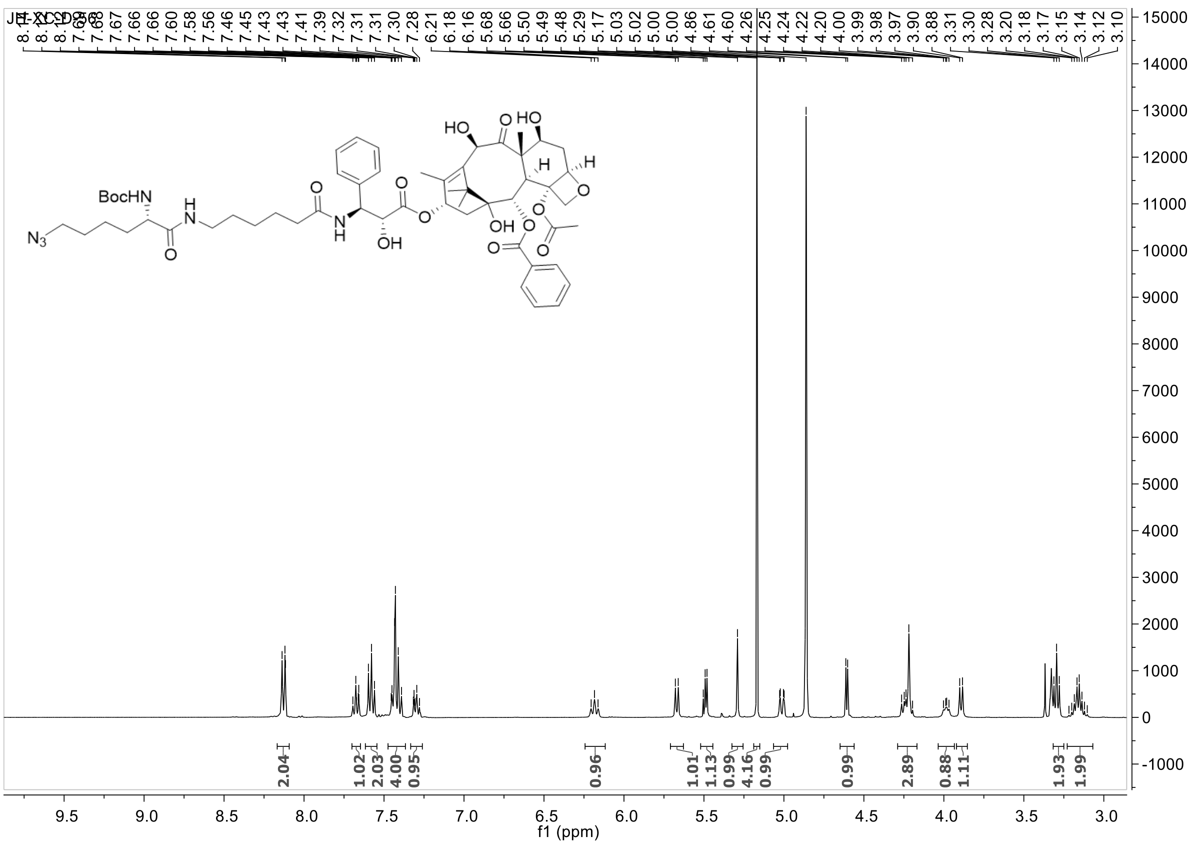
BocHN
N
H
N_3
O
OH
HO
H
f1 (ppm)

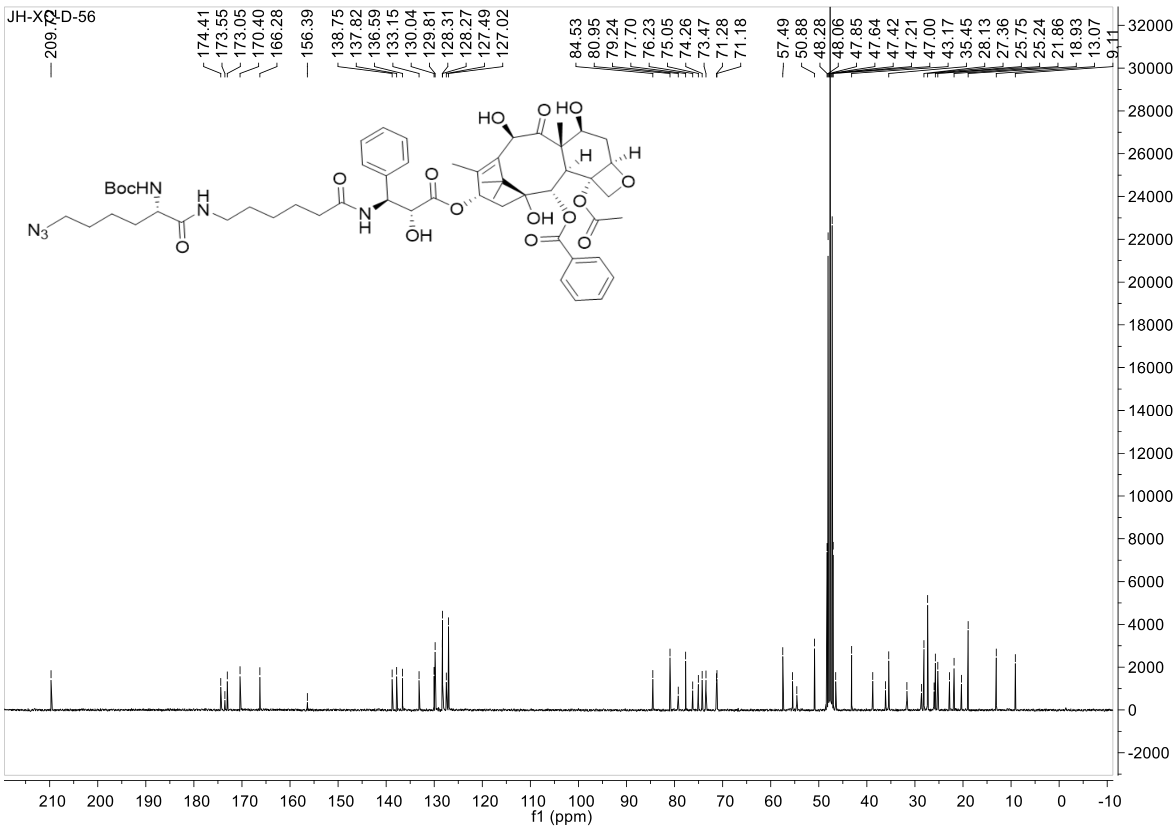

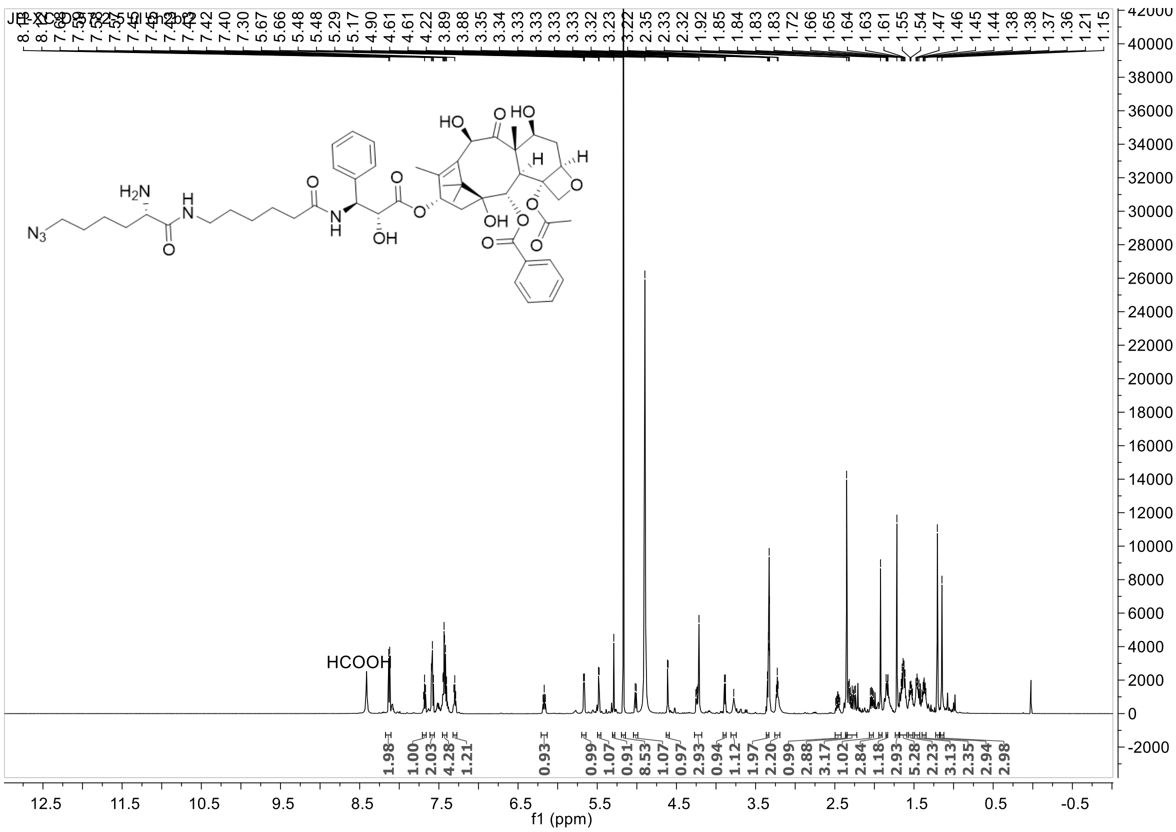

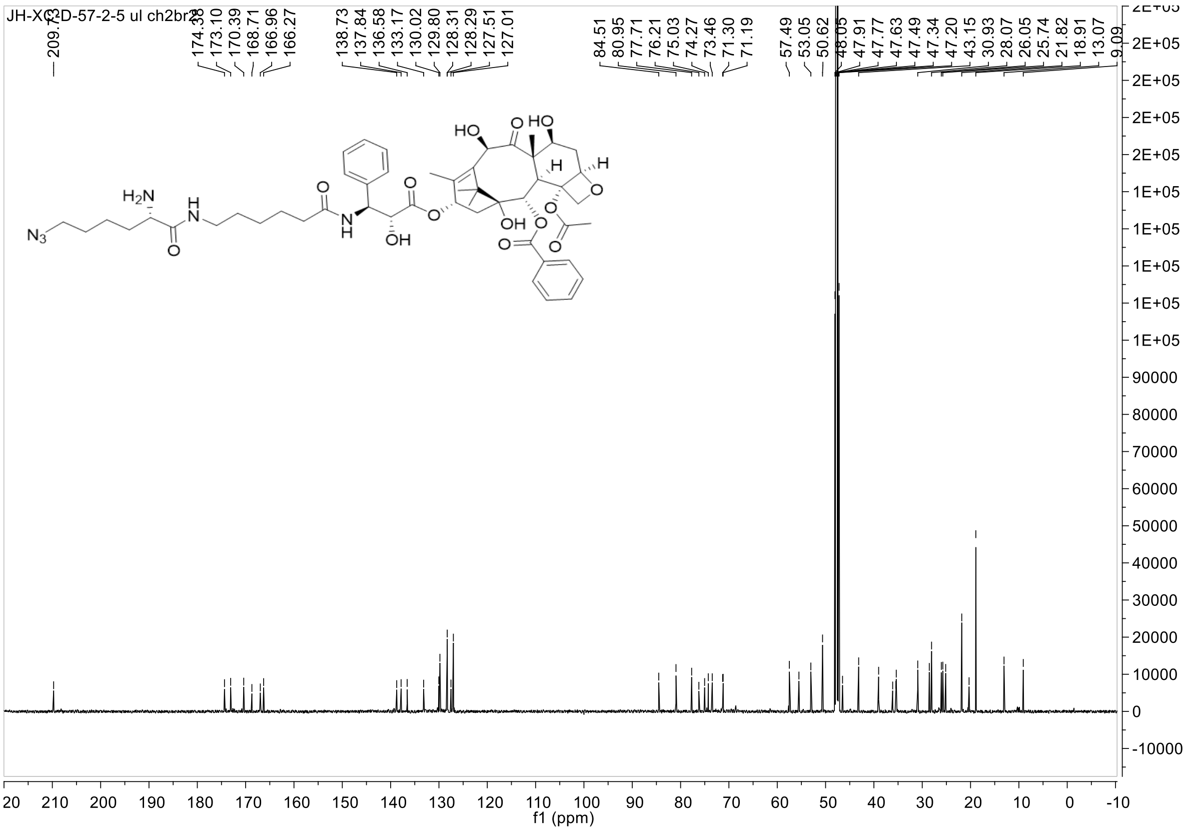

Reference

(1) Jain, D. R.; Ganesh, K. N. Clickable Cγ-Azido(Methylene/Butylene) Peptide Nucleic Acids and Their Clicked Fluorescent Derivatives: Synthesis, DNA Hybridization Properties, and Cell Penetration Studies. *J. Org. Chem.* **2014**, *79* (14), 6708–6714.
